# PumpKin: A machine-learning pipeline for automatically tracking localized kinematics in freely moving *C. elegans*

**DOI:** 10.1371/journal.pcbi.1014489

**Published:** 2026-07-17

**Authors:** Erin Shappell, Debra Buggs, Jennah Walcott, Hang Lu

**Affiliations:** 1 Interdisciplinary Program in Bioengineering, Georgia Institute of Technology, Atlanta, GeorgiaUnited States of America; 2 School of Electrical and Computer Engineering, Georgia Institute of Technology, Atlanta, GeorgiaUnited States of America; 3 School of Chemical & Biomolecular Engineering, Georgia Institute of Technology, Atlanta, GeorgiaUnited States of America; 4 Coulter Department of Biomedical Engineering, Georgia Institute of Technology, Atlanta, GeorgiaUnited States of America; Dartmouth College, UNITED STATES OF AMERICA

## Abstract

One of the many goals of neuroscience is to understand how the brain encodes and transforms sensory information into behavior. These animal behaviors can be studied at the level of multi-limb poses or through the focused analysis of individual body parts. Techniques for tracking animal pose, such as DeepLabCut and SLEAP, enable detailed studies of large-scale multi-limb behaviors but show reduced accuracy when used for single-keypoint tracking, where insufficient spatial context leads to increased drift and instability in tracking (Arent I, Schmidt FP, Botsch M et al. Marker-less motion capture of insect locomotion with deep neural networks pre-trained on synthetic videos. Frontiers in Behavioral Neuroscience. Vol. 15. 2021. Tang G, Han Y, Sun X, et al. Anti-drift pose tracker (ADPT), a transformer-based network for robust animal pose estimation cross-species. eLife. Vol. 13. 2025). More general techniques, such as Faster Region-based Convolutional Neural Network (Faster R-CNN) and You Only Look Once (YOLO), have also been used to track location-based behaviors such as center-of-mass position and velocity. However, behaviors localized to a single body structure, such as the pharyngeal pumping (i.e., feeding) in the microscopic roundworm *Caenorhabditis elegans* (*C. elegans*), are particularly sensitive to noise from moving non-target body parts. This limitation cannot be resolved by simply adding more training data, as doing so often leads to overfitting rather than improved robustness, and instead requires additional processing beyond existing object tracking packages. To address these challenges, we present a fast, automated method that reliably measures pumping in freely moving *C. elegans* by combining a state-of-the-art object detector (Faster R-CNN) with a tunable noise filter in a technique we call PumpKin. To validate its performance, we demonstrate both its speed (average of 0.4 seconds/frame) and its robust estimation capabilities through application to eight different experimental conditions that encompass both satiety and genetically-driven changes to feeding. PumpKin accurately estimates average pumping rates under eight different experimental conditions, which are positively correlated with the estimates of two expert annotators. Furthermore, PumpKin provides reliable estimates of the instantaneous pumping rate dynamics, achieving an average overlap that exceeds the human–human agreement measured via leave-one-out analysis. Applying PumpKin to conditions differing in satiety revealed a shared basal pumping rate of 0.5 Hz across all worm groups recorded off food, regardless of genetic background or satiety state. Together, these findings highlight PumpKin’s ability to accurately isolate and estimate the motion of a single body part during locomotion. Although we present results specific to *C. elegans*, we anticipate that PumpKin will generalize to behaviors localized to a single body structure in other systems.

## Author summary

Studies in neuroscience often seek to connect neural representations of sensory information with a corresponding behavioral response. These measured behaviors may be divided into two groups: those that involve multiple limbs and are easy to measure (e.g., locomotion) and those that involve only a single limb or organ and are more difficult to measure (e.g., swallowing food). Artificial intelligence (AI) has enabled the detailed study of these gross and localized behaviors. However, these technologies alone can struggle to track certain behaviors that are localized to a single limb or organ as a result of the behaviors’ high sensitivity to noise. One example of a localized behavior is found in the microscopic nematode *C. elegans*, where a pharyngeal organ called the grinder is measured to estimate the pumping (feeding) rate of the worms. The grinder is < 30 μm wide, less than the width of the average human hair, and moves at a speed of 5 pumps per second, equivalent to five times the average human heart rate. Thus, tracking this organ requires not only a sophisticated tracker, but also a program that can remove noise from the measured position of the grinder. To address this need, we introduce PumpKin: an open-source package designed to reliably measure the pumping rate of *C. elegans*.

## Introduction

Behaviors that are localized to a single body structure are critical to our understanding of larger behavioral patterns and decision-making. For example, the timing of blinking, although often perceived as spontaneous, is crucial for maintaining healthy vision [1–3]. Although comprehensive and generalizable toolboxes have been developed for larger-scale, multi-limb behaviors [4–9], these techniques cannot isolate the motion of a single body part from the organism’s full body locomotion or any external camera motion. This presents a challenge for studies that focus on the dynamics of individual body parts. Existing methods for tracking single-structure localized behaviors are often specialized to a single organism and restricted by specific experimental setups [10–13]. Although these methods excel in their intended applications, a general approach is still needed that can capture fine-scale, localized behaviors in freely moving animals without relying on specialized hardware or genetic modification.

**Fig 1:**
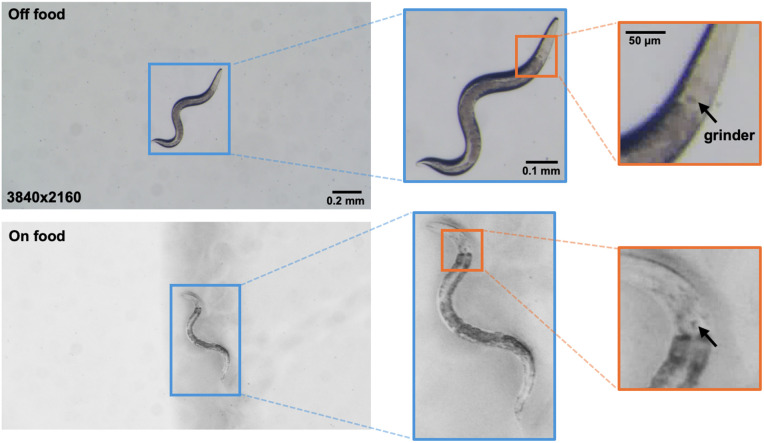
Representative examples of the worm and grinder in different environments. Contrast-enhanced images of worms both off (top row) and on food (bottom row) are shown. The original frames are shown on the left, with insets showing the worm (middle) and grinder (right) more clearly.

One such case of localized single-structure behavior involves the soil-dwelling nematode *Caenorhabditis elegans* (*C. elegans*). The optical transparency of *C. elegans* enables the direct measurement of feeding by recording the motion (i.e., pumping) of the grinder: a pharyngeal structure that processes food for digestion. Like many seemingly spontaneous behaviors in other organisms, such as blinking, eye saccades, and grooming, the pumping rate changes dynamically depending on several factors, including satiety and food availability [14]. However, measuring the pharyngeal pumping rate is challenging because the grinder is difficult to track. Its appearance varies due to full-body movements, changes in the shape of the grinder during pumping, and changes in contrast due to lighting distortions introduced by the surrounding bacteria lawn (Fig 1). These sources of variability make it difficult to obtain consistent measurements of the grinder during pumping. By addressing these challenges, we can establish a general framework for analyzing other localized single-structure behaviors that are similarly constrained by noise, motion, and variability.

Existing techniques for measuring the pharyngeal pumping rate are limited in their reliability, as manual methods are prone to human error and automated methods often perturb feeding (Table 1). Manual methods are considerably time-consuming, as pumps are counted by eye and often require slowing videos 2-4x for pumps to be visible and accurately counted by labelers. Manual counting is the standard for validating automated methods, including the method presented in this work, but variability among annotators makes it difficult to define a reliable baseline (S1 Fig and S1 Table). Although existing automated methods reduce variability, they often do so at the cost of altering the behavior being measured. Simpler automated techniques, such as thresholding, are effective when the worms are fixed in place but break down when applied to freely moving worms (S2 Fig). Fixing the worms in place disrupts feeding behavior [21–23] and even head fixation alone inhibits feeding [24,25], thus limiting the reliability of fixation-based techniques. Recent approaches, such as PharaGlow [13], employ genetic modification to illuminate the pharynx, enabling tracking in freely moving worms and eliminating the need for fixation. However, both genetic manipulation and the light exposure required for illumination can alter the behavior of the worm, including the pumping rate [26–28]. Furthermore, genetic modification may not always be possible or desired, as crossing the PharaGlow reagents would require backcrossing several generations to remove background differences. As mentioned previously, popular pose tracking techniques such as DLC and SLEAP are also unreliable with tracking single structures, including the grinder (see Figure Supplement 1 in [29]). Thus, there remains a need for an automated, label-free pumping rate estimator for freely-behaving *C. elegans*.

**Table 1:** A summary of existing methods for measuring pharyngeal pumping.

Method	Automated	Non-invasive	Free-moving	Label-free	Source(s)
Manual Counting	No	Yes	Yes	Yes	[15,16]*, Others*
Electropharyngeogram	No	No	No	Yes	[17]
Entropy differencing	Yes	Yes	No	Yes	[18]
Intensity differencing	Yes	Yes	No	Yes	[19]
Bioluminescence	Yes	Yes	Yes	No	[20]
PharaGlow	Yes	Yes	Yes	No	[13]
**PumpKin**	**Yes**	**Yes**	**Yes**	**Yes**	***This work***

Existing methods for measuring pharyngeal pumping are limited by the physical constraint of the worm, the use of fluorescent labels, and/or the genetic manipulation of the worm. Note that the phrase “label-free” is used to describe fluorescent labeling.

In this work, we introduce PumpKin: a non-invasive method for automatically tracking the localized kinematics of pharyngeal pumping in freely moving *C. elegans* that is fluorescent label-free. This algorithm combines Faster Region-based Convolutional Neural Network (Faster R-CNN) tracking with sparse optical flow to obtain instantaneous and average rate estimates for individual animals. We validate and demonstrate PumpKin in our application to track *C. elegans* pharyngeal pumping rate in brightfield. For this application, PumpKin utilizes our accessible all-in-one EZ-FRCNN package to quickly train and validate two Faster R-CNN models that work in tandem to track the pharyngeal bulb and the grinder, respectively [29]. Using a machine learning approach, such as Faster R-CNN, enables the reliable tracking of single structures, like the grinder, maintaining accuracy despite changes in shape, contrast, or lighting during freely moving experiments. This reliability is demonstrated by our successful application of PumpKin to several feeding conditions that represent different genetic backgrounds, satiety conditions, and recording conditions.

## Results

### Overview of the PumpKin pipeline

Tracking the motion of the grinder within a moving worm is challenging because the observed motion arises from several sources: the grinder itself, the worm, and plate movement to keep the worm in view. To remove plate motion, we first apply a Faster R-CNN model to extract a region of interest (ROI) that is centered on the portion of the pharynx containing the grinder and moves dynamically with the worm. A second Faster R-CNN model then detects the grinder within this ROI. To filter out remaining noise arising from worm movement, we use motion compensation and sparse optical flow to separate the grinder’s motion from external sources. Finally, to obtain a pumping rate from the tracked grinder motion, we detect pump event times using a biphasic peak detector and subsequently bin and smooth the event times. This multi-step approach enables robust isolation of grinder-specific motion despite organism-level and plate-level movement.

**Fig 2:**
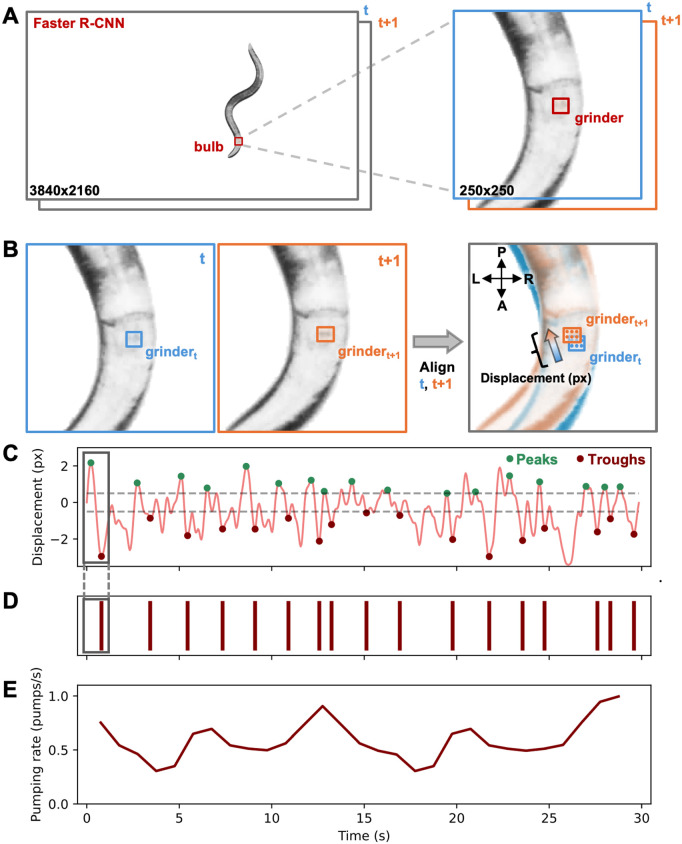
PumpKin uses Faster R-CNN, sparse optical flow, and biphasic peak detection to estimate the pumping rate. **(A)** The pharyngeal bulb of a worm is tracked in the original 3840 × 2160p video. A 250 × 250p region of interest (ROI) containing the grinder is cropped and analyzed using Faster R-CNN. **(B)** Consecutive frames (t, t + 1) are motion-compensated, and sparse optical flow is computed on sampled points within the grinder to estimate motion. **(C)** The grinder displacement is filtered and plotted over time. Pumps are detected via biphasic peak detection: green for anterior (peaks), red for posterior (troughs) motion. Dashed lines represent the spatial threshold for pump detection. The blue box denotes an example of a detected pump. **(D)** Pumps, defined by adjacent trough-peak pairs, are visualized as a raster plot. **(E)** The raster plot is binned and smoothed to estimate the instantaneous pumping rate.

To begin this pipeline, we implemented a preprocessing Faster R-CNN model that crops a ROI prior to tracking the grinder (Fig 2A). This step offers two key advantages: reduced processing time in later steps of PumpKin and improved tracking accuracy. The model first detects a larger body region containing the body part and then uses the center of the tracked bounding box to crop each video frame to contain the body part of interest. A second Faster R-CNN model is subsequently applied to track the body part of interest with high efficiency and precision (Fig 2B). This sequential use of two Faster R-CNN models generalizes readily to any animal or body part and enhances the overall performance of PumpKin.

Following Faster R-CNN tracking, we applied motion compensation to align adjacent frames, thereby reducing noise introduced by full-body movement and resulting in a signal that represents the grinder’s motion throughout the video (Fig 2B). From the motion-compensated frames, optical flow was used to estimate inter-frame motion of the grinder. The resulting motion signal was further refined with a low-pass filter, heuristically tuned to the data, to remove high-frequency noise. Users can estimate a cutoff frequency for this low-pass filter empirically based on the expected pumping rate for the genetic strain in the recording environment (e.g., on food or off food). For each experimental condition used in this study, the expected pumping rate was first determined empirically by an expert manual estimator, doubled to prevent aliasing, and used as the cutoff frequency.

In the final stage of the pipeline, we applied a biphasic peak detector to extract pump event times from the tracked grinder motion (Fig 2C and 2D). By defining the target motion profile and applying spatial and temporal filtering, we minimized residual artifacts and excluded parts of the signal unlikely to represent pump events. In the context of pharyngeal pumping, a single pump event is represented by a peak-trough pair in the grinder motion signal. Spatial filtering is completed by selecting a minimum and maximum valid peak (and trough) height. The user can determine these boundaries empirically or via a parameter sweep and will vary across imaging setups. These two parameters are generally insensitive, particularly in cases of low pumping (<3 pumps per second), and therefore require less tuning than the cutoff frequency (S5 Fig). Using the same cutoff frequency as the prior low-pass filter, temporal filtering excludes peak–trough pairs whose spacing is inconsistent with biophysically valid pumps. From the resulting detections, we calculated a continuous estimate of the pumping rate by binning and smoothing the pump event times (Fig 2E). The runtimes for all steps, including optional preprocessing steps are summarized in Table 2. A detailed parameter sweep for the cutoff frequency and minimum/maximum peak heights is summarized in S4 Fig, and precision-recall curves for the biphasic peak detector are provided in S5 Fig. The parameter values used for each experimental condition are provided in S2 Table

**Table 2:** The average runtime per frame for each processing step of the PumpKin pipeline.

Processing step	Average runtime (ms)
Faster R-CNN (4K video)*	211
Faster R-CNN (250x250p video)*	68
Dynamic video cropping*	28
Peak detection and continuous rate estimation	2

An asterisk* denotes an optional step. Runtimes were calculated by processing (40) 30-second videos, each with an average of 650 frames. All required steps were tested on the 250x250p cropped videos used for pump tracking.

### PumpKin reliably estimates the average pumping rate under challenging imaging conditions

We first validate PumpKin’s ability to capture known differences in the average pumping rate, such as those due to genetic mutation. To accomplish this, PumpKin was applied to 160 30-second videos of individual worms under eight experimental conditions, and a subset of 40 videos was manually annotated and used for validation. These conditions include changes in food availability during recording (on vs. off food), satiety before recording (fed vs. starved), and genetic background (N2 vs. *eat-2*). The gene *eat-2* encodes a nicotinic acetylcholine receptor whose function is necessary for rapid pharyngeal pumping [30,31]. The mutant *eat-2* experiences a much lower average pumping rate (1–2 pumps/second) than that observed in the control background N2 (4–5 pumps/second) [20,30,32,33] and provides a suitable test case to track altered pumping rates on and off food. The food and satiety conditions were included to showcase PumpKin’s ability to generalize across changes to the environment and worm behavior.

**Fig 3:**
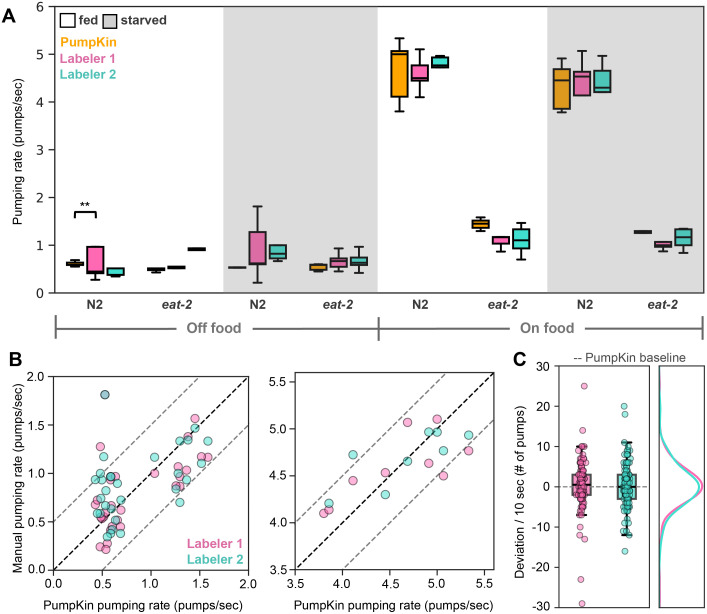
PumpKin generates pumping rate estimates consistent with those obtained by expert manual labelers and that capture known differences in pumping due to satiety and genetic mutation. **(A)** Average pumping rates estimated by PumpKin are compared with those obtained by two manual labelers of high expertise. N = 5 per box. Statistical significance was determined via unpaired t-test: *p < 0.05, **p < 0.01, ***p < 0.001 **(B)** Comparison of the average pumping rates estimated by PumpKin and two expert labelers, separated by low (0-2 pumps/sec) and high (3.5-5.5 pumps/sec) pumping rates. The center dashed line represents perfect correlation, and the additional dashed lines represent perfect correlation shifted by ±0.5 pumps/sec. **(C)** Deviation in pump count between PumpKin and expert estimates within a 10-second clip. 30-second videos were divided into (3) 10-second video clips and pump count discrepancies were calculated by subtracting the PumpKin counts from the expert counts. N = 120 per bar. Dashed line represents PumpKin baseline. KDE distributions for each labeler are shown on the right.

Compared to expert manual annotations, PumpKin produced similar average pumping rate estimates across all eight experimental conditions (Fig 3A). This agreement was supported quantitatively by unpaired t-tests, which revealed few significant differences between estimates generated by PumpKin and those from manual labelers. Consistent with previous reports, PumpKin estimated an on-food average pumping rate of 1.4 pumps/second for *eat-2* and 4.7 pumps/second for N2. Average pumping rates for all experimental conditions are summarized in Table 3. Taken together, these results indicate that PumpKin reliably measures the average pharyngeal pumping rate in freely moving worms.

**Table 3:** PumpKin’s estimated average pumping rate per experimental condition.

Experimental Condition	Pumping rate (pumps/s)
N2 fed → no food	0.54 ± 0.11
*eat-2* fed → no food	0.54 ± 0.12
N2 starved → no food	0.54 ± 0.08
*eat-2* starved → no food	0.54 ± 0.10
N2 fed → food	4.71 ± 0.34
*eat-2* fed → food	1.38 ± 0.18
N2 starved → food	4.44 ± 0.35
*eat-2* starved → food	1.35 ± 0.15

N = 20 per condition.

To further validate PumpKin, we compared its average pumping rate estimates and total pump counts with those obtained by manual annotation. We found that PumpKin’s average estimates for both low (0–2 pumps/sec) and high (3.5-5.5 pumps/sec) pumping rates were positively correlated with those obtained by two expert manual labelers (Fig 3B and S3 Table). Total pump counts were compared by dividing all videos into 10-second intervals and subtracting the number of pumps labeled by PumpKin from the number of pumps labeled by each manual labeler to obtain a pump count deviation (Fig 3C). Both labelers show an average pump deviation close to 0 compared to PumpKin, further supporting PumpKin as a reliable estimator for the average pumping rate. Together, these results show that PumpKin can generate average pumping rates comparable to those obtained by experienced human annotators.

### PumpKin reliably estimates the instantaneous pumping rate

In addition to validating PumpKin’s average pumping rate estimates, we next evaluated its ability to capture instantaneous pumping dynamics within the 30-second recording period. The pharyngeal pumping rate is modulated through “bursts” and pauses in pumping [19] depending on the concentration of bacteria present. While important, instantaneous pumping has been less studied, as most pump measurement methods record only the average pumping rate over an interval rather than an instantaneous rate. Using the same validation set (N = 40 videos), we compared PumpKin’s instantaneous pumping rate estimates with those generated by manual labelers of varying expertise to determine whether PumpKin could correctly identify changes in pumping dynamics.

To quantify how well PumpKin’s instantaneous estimates agreed with those generated by manual labelers, we calculated the percentage of the signal for which PumpKin’s estimate fell within a data-driven tolerance derived from human variability. For each video, we defined the consensus across manual labelers as the median of their pumping rate estimates. Then, the standard deviation (SD) across each manual labeler’s difference from the consensus was used to define the confidence interval (± 1.5 pooled SD). The width of the confidence interval varies across videos, with narrower intervals corresponding to higher agreement among the three labelers. The overlap between PumpKin’s estimate and the confidence interval was calculated as the fraction of timepoints in which PumpKin’s estimate fell within the bounds. To provide a more direct comparison between the performances of PumpKin and the manual labelers, we also calculated a leave-one-out overlap for each of the three manual labelers.

**Fig 4:**
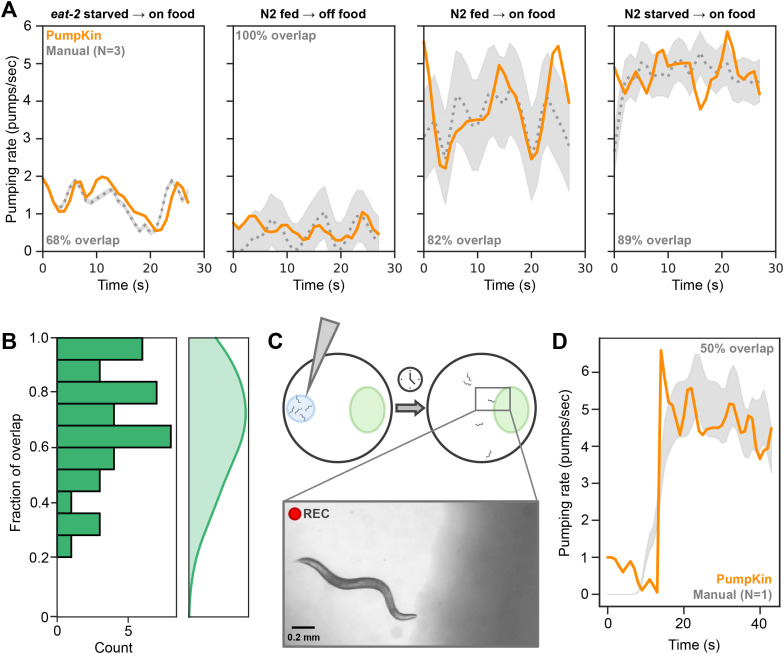
PumpKin generates instantaneous pumping rate estimates consistent with those obtained by manual labelers. **(A)** Representative examples of instantaneous pumping rate estimates from PumpKin compared to manual estimates (N = 3 labelers) from four out of the eight experimental conditions. The shaded region represents the range of manual estimates (± 1.5 pooled SD), and the percent of the PumpKin signal that overlaps with this region is noted in each example. The dashed line denotes the consensus (median) of the three manual estimates. **(B)** The distribution of overlap between PumpKin instantaneous estimates and the range of manual instantaneous estimates (± 1.5 pooled SD) across all videos used for validation (N = 40). The KDE distribution is shown on the right. **(C)** An additional experiment that measured the pumping rate during lawn entering (i.e., transition from off food to on food) was completed to test PumpKin’s ability to capture a low-to-high pumping rate transition. **(D)** The instantaneous pumping rate estimate of a single worm entering the lawn compared to a single manual estimate (± 15% tolerance).

Qualitatively, most of PumpKin’s estimates fall within or close to this confidence interval across all experimental conditions, with some specific examples shown in Fig 4A. Across all 40 videos, PumpKin’s estimates overlapped an average of 69% with manual estimates (Fig 4B), while the average human-human leave-one-out overlap was 59% ± 11%. These results demonstrate that PumpKin’s estimates reliably match or exceed manual performance. Overall, these results suggest that PumpKin not only provides reliable average estimates, but also reliable estimates of the dynamics.

Building on these results, we further investigated whether PumpKin could be adapted to estimate significant changes in pumping dynamics. In decision-making and feeding experiments, animals are not always confined to strictly food or non-food spaces, which can introduce substantial variability in feeding rate. To test whether PumpKin can be modified to capture these larger increases in pumping rate, we recorded a single worm entering a lawn after being moved onto a new plate (Fig 4C). Because the low-pass filter designed for noise removal requires different parameters based on the expected pumping rate, we exploited the predictability of the worm’s pumping rate increase upon lawn entering and used separate parameters for the pre-lawn entry (0–14 seconds) and post-lawn entry (14–44 seconds) pumping rates. For this recording, annotations were obtained from a single manual labeler, and the confidence interval is therefore defined as a fixed ±15% tolerance. The resulting estimate is consistent with that of a single expert estimator, with an overlap of 50% (Fig 4D).

### PumpKin identifies differences in pumping rate driven by satiety

**Fig 5:**
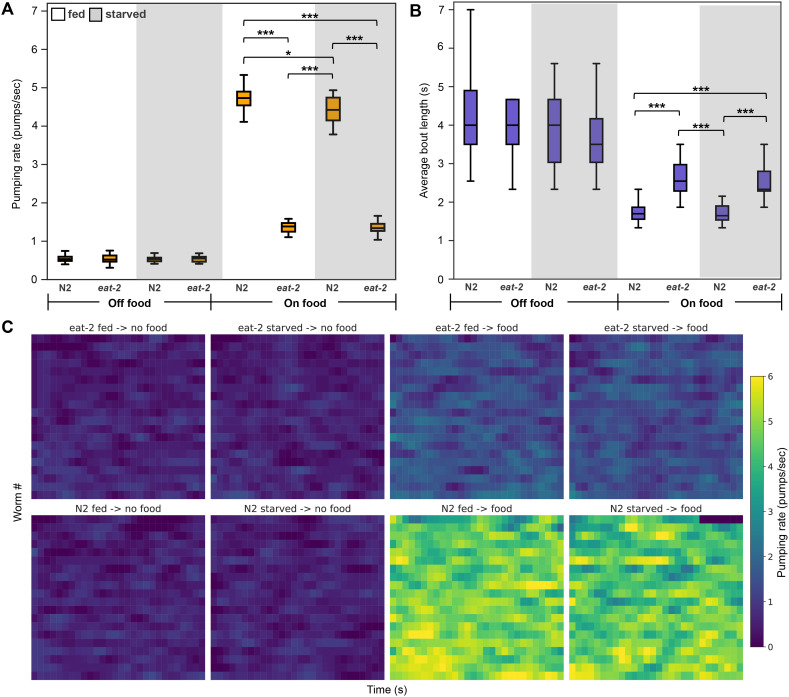
PumpKin’s estimates of the pumping rate reveal a condition-agnostic decrease in the off food pumping rate. **(A)** PumpKin estimates of the average pumping rate across 160 videos of individual worms. N = 20 per box. Pairwise statistical significance was determined via unpaired t-test: *p < 0.05, **p < 0.01, ***p < 0.001. **(B)** The average bout length across all eight experimental conditions. **(C)** Heatmap summary of the instantaneous pumping rates for all 160 worms. The time axis spans 28 s with 1 s bins. Heatmaps are organized by condition, and pumping rates are organized via Principal Component Analysis (PCA) for readability. Note that the first trace of the N2 starved → food condition is taken from a shorter video, thus the trace ends before the 28s interval.

Although feeding requires pumping, worms continue to pump even when food is absent [13,18,34–36]. This behavior is particularly of interest as the pumping rate dynamics may reflect active decision-making in the worm during exploration. The pumping rate of freely moving worms without food present is less studied due to the constraints of existing pumping rate measurement methods and the difficulties specific to counting pumps in the moving worm. Off-food pumping rates are known to change based on the amount of time spent off food. For example, early-phase starvation (≤120 minutes) results in an average pumping rate of 0.5-0.8 pumps/second [13,35], while late-phase starvation (>120 minutes) results in an average pumping rate of 1-1.3 pumps/second [35]. In this work, we investigated two early-phase starvation groups for each genetic background (N2, *eat-2*): one group that was fed for one hour and then transferred to a plate without food for recording (fed → off food) and another group that was starved for one hour and then transferred to a plate without food for recording (starved → off food). These two groups represent samples from the beginning and end of early-phase starvation, respectively. Our results agree with previous measurements of early-phase off-food pumping, as we report that all worms that were recorded off-food shared a low average pumping rate of 0.5 pumps/second, regardless of their previous satiety (Figs 3A and 5 and S4 Table).

On-food pumping is also modulated based on the prior satiety of the worm. After longer periods of starvation (>6 hours), worms exhibit faster pumping upon reintroduction to food [35]. In cases of shorter starvation, such as those represented by the starved → on-food condition in this study, worms will increase their pumping rate more gradually [35,37]. We found that N2 worms that were reintroduced to food post-starvation had a slightly lower pumping rate than worms that were fed before recording (Fig 5A), with well-fed on-food N2 pumping being about 0.27 pumps/second higher. Interestingly, *eat-2* worms under the same conditions did not show a significant difference in pumping. This result likely reflects the impairment of the cholinergic pathway in *eat-2*, which mediates fast pumping and is further activated upon reintroduction to food following starvation. [19,35].

Interestingly, we observed both slow and fast pumping bouts within the 30-second recording period of multiple worms (Fig 5B and 5C). These patterns are most visible in the N2 on-food groups, but also appear in the other experimental conditions. Although a small selection of the slow pumping bouts represents short grinder occlusions, the existence of these bouts may suggest that higher pumping rates are less uniform than they appear. To quantify this phenomenon, we applied run-length encoding (RLE) to each worm’s pumping rate to split the continuous signal into bouts of similar pumping rates (within 0.25 pumps/second). The bout length acts as an estimate of the homogeneity within the signal; a longer bout length corresponds to a more homogeneous pumping rate.

We found that the average bout length was generally longer in off-food groups and shorter in on-food groups, independent of the genetic background (Fig 5B and S5 Table). This result is consistent with our previous finding that all off-food groups shared a similar “basal” pumping rate of 0.5 pumps/second, suggesting that worms off food do not change their pumping rate as often as worms on food. Furthermore, the *eat-2* on-food bout lengths were consistently longer than those of N2 on food. This suggests that, in addition to having a lower pumping rate than N2, *eat-2* worms also adjust their pumping rate less frequently, even in the presence of food. Overall, these findings enrich our understanding of how both the *eat-2* genetic background and the presence of food each affect the pumping rate dynamics as well as the average pumping rate.

## Discussion

Here, we introduced PumpKin: an automated method for tracking pharyngeal pumping, a challenging yet well-characterized behavior essential for *C. elegans* feeding. By combining several generalizable noise reduction steps, PumpKin enables detailed behavioral measurements that surpass what is achievable with widely used tracking approaches. We validated PumpKin’s performance through the measurement of eight experimental conditions that encompass a range of satiety and genetically-driven changes to the pumping rate. However, this validation was performed on a relatively small number of samples (5 per condition, 40 total). This limitation reflects the time investment required for a detailed manual annotation of pumping, which took approximately 2–6 minutes per sample depending on the sample’s difficulty. In addition, we did not directly compare PumpKin’s results to alternative automated tracking approaches such as PharaGlow, which may limit conclusions about PumpKin’s relative performance. Although comparison to approaches requiring specialized genetic strains and/or instrumentation would be informative, we emphasize that manual annotation remains the primary ground truth in measuring pumping and thus provides a meaningful basis for evaluation. Despite the limited sample size and lack of direct comparison to this competing method, the strong agreement observed between PumpKin and manual annotations across all conditions supports the robustness of the approach.

Through the application of PumpKin to our full dataset (20 samples per condition, 160 total), we revealed differences in the finer-scale behavioral dynamics across both satiety and genetically driven differences in pumping. Using RLE to quantify pumping rate homogeneity, we showed that worms off food exhibit more homogeneous pumping than worms on food. Moreover, *eat-2* mutant worms demonstrated more homogeneous pumping rates than N2 worms while on food, consistent with this mutant’s impaired rapid pumping in the presence of food. Future work examining pumping rate dynamics in additional genetic backgrounds (e.g., wild isolates, other feeding mutants) may extend these findings and further elucidate how genetic variation shapes the regulation of *C. elegans* feeding.

These results highlight the advantages of PumpKin, which has enabled the first measurements of pumping in *C. elegans* without fixation or transgenic reporters. By leveraging the Faster R-CNN framework and tunable noise filtering, this package allows researchers to incorporate pharyngeal pumping into detailed behavior analyses with minimal modifications to existing imaging setups. Because fluorescence reporters are not required for pumping measurement, PumpKin can be combined with measurements of gene expression or, eventually, neuronal activity, allowing for the simultaneous characterization of feeding and circuit-level dynamics. Furthermore, although most *C. elegans* feeding studies are completed using thin, uniform bacterial lawns, the flexibility of the Faster R-CNN framework allows this pipeline to learn the appearance of the grinder in more heterogeneous environments that more closely resemble the worm’s natural habitat (e.g., rotting fruit). In general, PumpKin is adaptable to diverse environments and genetic backgrounds, facilitating future work on how feeding varies across behavioral and ecological conditions.

Outside of our application to pharyngeal pumping in *C. elegans*, the PumpKin pipeline can easily be adapted and applied to track various localized behaviors in other organisms, such as pupilometry in rodents. While we utilized our all-in-one Faster R-CNN pipeline, EZ-FRCNN, for tracking the grinder, the motion compensation and biphasic peak detection are applicable to results of other object trackers such as YOLO (You Only Look Once) [38] and DETR (Detection Transformer) [39], which may be used to track other behaviors. Our findings demonstrate that PumpKin can reveal subtle variations in the frequency of a behavior and how these changes are shaped by environmental and genetic factors. Therefore, these results highlight PumpKin as a versatile tool that enhances behavioral tracking by enabling the detection of subtle but biologically significant patterns across species.

## Materials and methods

### *C. elegans* maintenance

The *C. elegans* strains used in this work include N2 and DA1113 [*eat-2 (ad1113)*]. All *C. elegans* strains were cultured on Nematode Growth Media (NGM) plates at 20° C. Day 1 adults were obtained by a six-hour hatch off for N2 and a three-hour hatch off for *eat-2* to account for its lower egg-laying rate. Two days later, the resulting age-synchronized adults of each strain were assayed.

### Plate preparation

To maintain a consistent environment between experiments and experimental groups, all plates were prepared using the same batch of agar and OP50 bacteria. The plates used for each experiment were seeded with 200 µL of OP50 (cultured overnight in LB) two days before the experiment to ensure consistent food quality.

### Feeding assays

To prepare worms for the 1-hour starvation/feeding period, each strain was washed off 1X with 1 mL S-basal into a centrifuge tube. The worms were then centrifuged for 1 minute, and the supernatant was removed by pipette. The worms were rewashed with 1 mL S-basal, mixed for 1 second and then centrifuged again for 1 minute to remove any remaining bacteria from the initial wash. After removing the supernatant from the second wash, half of each washed strain was pipetted onto a plate without food and the other half was pipetted onto a plate with OP50 bacteria. After 1 hour, all worms were washed off following the same procedure noted previously. After washing, the starved and fed group in each strain was pipetted onto two recording plates: half onto a plate with food and half onto a plate without food. This procedure is summarized visually in (S3 Fig).

Recordings were completed using a stereomicroscope (Leica M165 FC) with an 8.3-megapixel camera (InfinityCam 8–8). For each recording, five worms were randomly selected from each experimental group and individually recorded for 30 seconds. Four total recording sessions were completed for a total of 20 unique worms in each experimental group. Videos were recorded in 3840x2160 resolution at 6.3x magnification for 30 seconds at an acquisition rate of 22 FPS. In cases where the worms moved significantly (e.g., worms recorded off food), the plate was manually moved to prevent the worm from leaving the camera’s field of view.

### Obtaining unbiased manual estimates of pharyngeal pumping

To validate PumpKin’s pumping rate estimates, we had three labelers with varying levels of expertise complete double blind counts for 1/4 of the dataset (i.e., one recording session). Manual labelers were ranked according to their pump count experience, the highest ranked labeler having four years of pump counting experience and the lowest ranked labeler having several years of experience working with *C. elegans*, but less than one year of pump count experience. Pumps were counted at the end of the pump cycle and pump times were recorded by clicking a computer mouse. Each labeler recorded the pump event times to obtain both an instantaneous pumping rate estimate and an overall average. All labelers recorded pump event times at the end of each pump, with each video slowed by 4x to ensure consistency across labelers.

### Training the faster R-CNN networks

Faster R-CNN model training was completed using our all-in-one Faster R-CNN package, EZ-FRCNN [29]. This package uses PyTorch’s torchvision package (version 0.17.0) and the ResNet-50-FPN model architecture. Each model was initialized using COCO v1 weights and then trained on our data for 50 epochs using an NVIDIA GeForce RTX 3070 Ti Laptop GPU. A separate model was trained to track the pharyngeal bulb (i.e., the portion of the pharynx containing the grinder, the structure of interest) first to obtain a region-of-interest centered on the putative grinder location. To train this model, a set of 250 frames was extracted from 11 videos using DeepLabCut’s k-means clustering method [7,8]. An 80/20 train/test split was used for model training. The resulting model was evaluated on a heldout set of 14 videos prior to using the model to obtain a cropped (250x250p) ROI centered on the pharyngeal bulb.

A second Faster R-CNN model was trained to track the grinder by extracting 100 training images from the ROIs of the same 11 videos used to train the first model. The same k-means method was used to sample these frames, and the same 80/20 train/test split was used for training. The resulting model was evaluated using the ROIs of the same heldout set of 14 videos used for validating the pharyngeal bulb-tracking Faster R-CNN model.

All training frames were labeled manually using an open-source labeling tool [40]. Additional details and results from model training, testing, and cross-validation can be found in [29]. Example videos of individual worms with the pharyngeal bulb and grinder tracked by Faster R-CNN are provided in S1 Video-S9 Video.

### Dynamic cropping of a region-of-interest (ROI) for noise reduction

To reduce noise from plate motion, a Faster R-CNN model [29] was trained to track the pharyngeal bulb: the part of the pharynx containing the grinder. The location of the pharyngeal bulb was then used to crop a 250x250 pixel region of interest (ROI) for each frame, reducing both noise and the processing power required to track the movement of the grinder (Fig 2A).

### Motion compensation for reduction of noise from body motion

After an ROI was dynamically cropped for the full video, motion compensation was applied to each cropped frame to reduce noise from worm movement. First, a Shi-Tomasi corner detector [41] was used to extract significant features from the current frame, including the edges of the grinder, the pharyngeal bulb, and the worm. These features were then matched to the previous frame and used to calculate flow vectors representing the motion of each feature from the previous frame to the current frame. Using Random Sample Consensus (RANSAC) [42], an affine transformation was estimated from the matched feature pairs to align each pair of adjacent frames. The final motion-compensated flow vectors were obtained by subtracting the aligned features in the previous frame from the matching features in the current frame (Fig 2B).

### Design of a custom Butterworth filter for high-frequency noise reduction

Next, a point-cloud representation of the grinder from the Faster R-CNN label was obtained by converting the original bounding box into a 15x15 pixel area centered on the grinder and uniformly sampling nine points from this area. Compensated motion vectors were calculated for each of the nine grinder points and averaged to obtain a stable estimate of the grinder motion for each pair of adjacent frames. These vectors were then converted into a time series by calculating the signed magnitude of each vector. A custom-fit second-order low-pass Butterworth filter was applied to the time series signal to remove any outstanding high-frequency noise prior to pump detection (Fig 2C, Eq 1).


$$\begin{array}{c} H(z)=\frac{b_{0}+b_{1}z^{-1}+b_{2}z^{-2}}{a_{0}+a_{1}z^{-1}+a_{2}z^{-2}} \end{array}$$ (1)

The filter coefficients ($a_{0},...,b_{2}$) are computed using the bilinear transform, based on the following frequency definitions:


$$\begin{array}{cc} \omega_{c} & =\text{cutoff frequency}\\ \omega_{s} & =\text{sampling frequency}\\ W_{n} & =\frac{\omega_{c}}{\omega_{s}}\;\text{(normalized cutoff frequency)} \end{array}$$

The cutoff frequency ($w_{c}$) for this filter was calculated based on the predicted pumping rate of the strain of interest and the Nyquist-Shannon sampling theorem. Then, we define the bilinear transform warping parameter:


$$\begin{array}{c} K=\tan\left(\frac{\pi W_{n}}{2}\right) \end{array}$$

Then, the filter coefficients ($a_{0},...,b_{2}$) are given by:


$$\begin{array}{cc} a_{0} & =K^{2}+\sqrt{2}K+1\\ a_{1} & =2(K^{2}-1)\\ a_{2} & =K^{2}-\sqrt{2}K+1\\ b_{0} & =K^{2}\\ b_{1} & =2K^{2}\\ b_{2} & =K^{2} \end{array}$$

### Design of a biphasic peak detector for pump event tracking

After obtaining a time series estimate for grinder motion, a biphasic peak detection algorithm was applied to identify pharyngeal pumps. During a pharyngeal pump, the grinder contracts and relaxes. Grinder contraction is visually represented by an anterior movement, while relaxation is represented by a posterior movement. Since a traditional peak detector only detects positive local maxima (peaks) in a signal, we combined two peak detectors: one to track peaks and one to track troughs by flipping the sign of the original signal. We then defined a pump as a peak followed by a trough (Fig 2D).

To further account for noise, two parameters were tuned for this peak detection algorithm: the maximum allowed distance between peaks and troughs, and the range of heights allowed for peaks and troughs to be considered pumps. The first parameter temporally filters peak-trough pairs that are too far apart to be considered pumps (i.e., motions that are too slow to represent a pump). The second parameter applies a spatial filter to remove peaks and troughs that are too small to be biologically meaningful and are likely noise. These values are tunable to the user’s dataset and may vary slightly depending on factors such as magnification and video resolution. The distance parameter is measured in frames and can be estimated by dividing the frame rate by the expected average movement rate of the structure of interest; for our example, we estimate this parameter by dividing the frame rate by the expected average pumping rate of the worm. The height parameter is measured in pixels and should be determined empirically.

### Use of peristimulus time histogram (PSTH) to estimate a continuous pumping rate from event times

Pump event times were calculated based on the end of each detected pump (i.e., the time at which the trough occurs). These pump times can then be binned and filtered using a peristimulus time histogram (PSTH) inspired technique to obtain an instantaneous estimate of the pumping rate for the full video (Fig 2E). Or, if the user prefers only an average estimate over a select interval, the pump times can simply be averaged over the interval to obtain a single average rate.

### Timing analyses

All timing analyses of the PumpKin processing steps outlined in Table 2 were completed using an NVIDIA GeForce 3070 Ti Laptop GPU. Runtimes were averaged over 40 videos sampled from the 160 video dataset presented in this work.

## Supporting information

S1 Fig Manual estimates of pumping rate are dependent on experience. (A) Average pumping rates obtained by three double-blinded manual labelers of varying expertise. Labelers are categorized by pump counting expertise, with Labeler 1 having the most experience and Labeler 3 having the least. N = 5 per box. Statistical significance was determined via unpaired t-test: *p < 0.05, **p < 0.01, ***p < 0.001. (B) Comparison of the condition-averaged pumping rates of Labelers 2 and 3 with those of Labeler 1, separated by low (0–3 pumps/sec) and high (3–6 pumps/sec) pumping rates. The center dashed line represents perfect correlation, and the additional dashed lines represent perfect correlation shifted by ±0.5 Hz. (C) Deviation in pump count between manual estimates within a 10-second clip. 30-second videos were divided into (3) 10-second video clips and pump count discrepancies were calculated by subtracting the PumpKin counts from the manual counts. N = 120 per bar. KDE distributions for each labeler pairing are shown on the right.
(TIF)

S2 Fig Simpler computational techniques, such as thresholding, do not consistently track the grinder in freely moving worms. Three sample frames obtained from a single video were thresholded using three popular thresholding techniques. The performance of thresholding the grinder is highly dependent on image quality, which can vary as the worm moves freely.
(TIF)

S3 Fig Eight total experimental conditions were studied by testing two satiety conditions, two recording conditions, and two genetic backgrounds. Each genetic strain is separated into two satiety groups: one that is starved for one hour and one that is fed. After 1 hour, each satiety group is further divided into two subgroups: one that is recorded on food and one that is recorded off food.
(TIF)

S4 Fig A parameter sweep for PumpKin’s cutoff frequency, minimum valid peak height, and maximum valid peak height across three experimental conditions. A parameter sweep was conducted for three of the eight experimental conditions discussed in the text, each with a different pumping rate: starved N2 worms off food (A), fed *eat-2* worms on food (B), and fed N2 worms on food (C). The results for each sweep were calculated as the mean error when compared to two expert manual labelers, where the mean across all samples (N = 5) is represented by a line and the min-max range is represented by a shaded region. Star denotes the parameter value used for each condition in this study.
(TIF)

S5 Fig Precision-recall analysis for PumpKin’s biphasic peak detector based on parameter sweeps over the cutoff frequency, minimum valid peak height, and maximum valid peak height across three experimental conditions. A precision-recall curve was provided for each parameter sweep for three of the eight experimental conditions discussed in the text, each with a different pumping rate: starved N2 worms off food (A), fed *eat-2* worms on food (B), and fed N2 worms on food (C). PumpKin’s estimated pump times were matched 1:1 to a consensus between the two expert manual labelers’ reported pump times within a time window Δt (A: Δt = 1.2, B: Δt = 0.7, Δt = 0.2). The final precision and recall values were calculated on the pooled counts across the samples in each experimental condition (N = 5). Star denotes the parameter value used for each condition in this study. Plus sign (+) indicates the labeler-labeler precision/recall.
(TIF)

S1 Video Example video of pharyngeal bulb tracking in a worm on a bacteria lawn. (AVI)

S2 Video Example video of grinder tracking in a worm on a bacteria lawn. (AVI)

S3 Video Additional example video of pharyngeal bulb tracking in a worm on a bacteria lawn. (AVI)

S4 Video Additional example video of grinder tracking in a worm on a bacteria lawn. (AVI)

S5 Video Example video of pharyngeal bulb tracking in a moving worm on agar. (AVI)

S6 Video Example video of grinder tracking in a moving worm on agar. (AVI)

S7 Video Additional example video of pharyngeal bulb tracking in a moving worm on agar. (AVI)

S8 Video Additional example video of grinder tracking in a moving worm on agar. (AVI)

S9 Video Video of grinder tracking in a worm approaching and entering the bacteria lawn. (AVI)

S1 Table All Pearson’s R values from comparing Labeler 1’s average pumping rates to those obtained by two additional labelers from S1 Fig. (XLSX)

S2 Table All PumpKin parameter values used for each experimental condition. (XLSX)

S3 Table All Pearson’s R values from comparing PumpKin’s average pumping rates to those obtained by two expert labelers from Fig 3B. (XLSX)

S4 Table All unpaired T-test results from comparing the PumpKin average rate estimates from Fig 5A. (XLSX)

S5 Table All unpaired T-test results from comparing the average pumping bout lengths from Fig 5B. (XLSX)
