# Supplementary figures and images for: PumpKin: A machine-learning pipeline for automatically tracking localized kinematics in freely moving C. elegans
Source: PLoS Comput Biol. 2026 Jul 17;22(7):e1014489. doi: 10.1371/journal.pcbi.1014489 (PMC13399524; doi:10.1371/journal.pcbi.1014489)

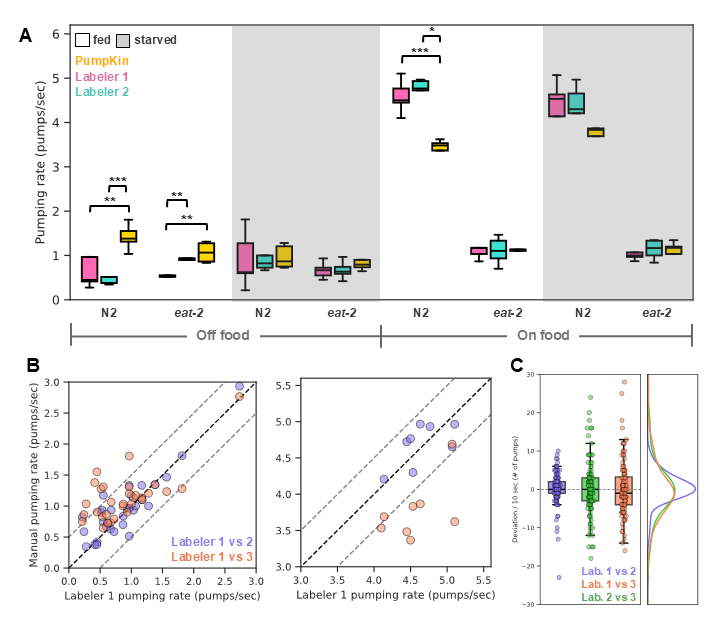

Supplement: S1 Fig — (A) Average pumping rates obtained by three double-blinded manual labelers of varying expertise. Labelers are categorized by pump counting expertise, with Labeler 1 having the most experience and Labeler 3 having the least. N = 5 per box. Statistical significance was determined via unpaired t-test: *p < 0.05, **p < 0.01, ***p < 0.001. (B) Comparison of the condition-averaged pumping rates of Labelers 2 and 3 with those of Labeler 1, separated by low (0–3 pumps/sec) and high (3–6 pumps/sec) pumping rates. The center dashed line represents perfect correlation, and the additional dashed lines represent perfect correlation shifted by ±0.5 Hz. (C) Deviation in pump count between manual estimates within a 10-second clip. 30-second videos were divided into (3) 10-second video clips and pump count discrepancies were calculated by subtracting the PumpKin counts from the manual counts. N = 120 per bar. KDE distributions for each labeler pairing are shown on the right. (TIF) [file pcbi.1014489.s001.tif]

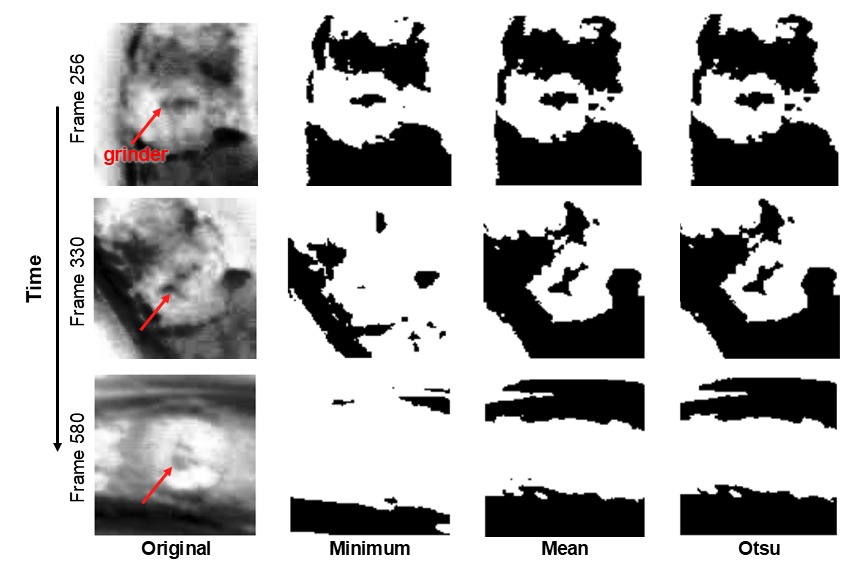

Supplement: S2 Fig — Three sample frames obtained from a single video were thresholded using three popular thresholding techniques. The performance of thresholding the grinder is highly dependent on image quality, which can vary as the worm moves freely. (TIF) [file pcbi.1014489.s002.tif]

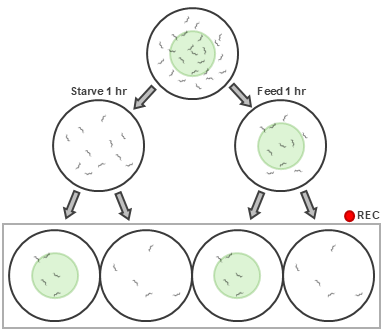

Supplement: S3 Fig — Each genetic strain is separated into two satiety groups: one that is starved for one hour and one that is fed. After 1 hour, each satiety group is further divided into two subgroups: one that is recorded on food and one that is recorded off food. (TIF) [file pcbi.1014489.s003.tif]

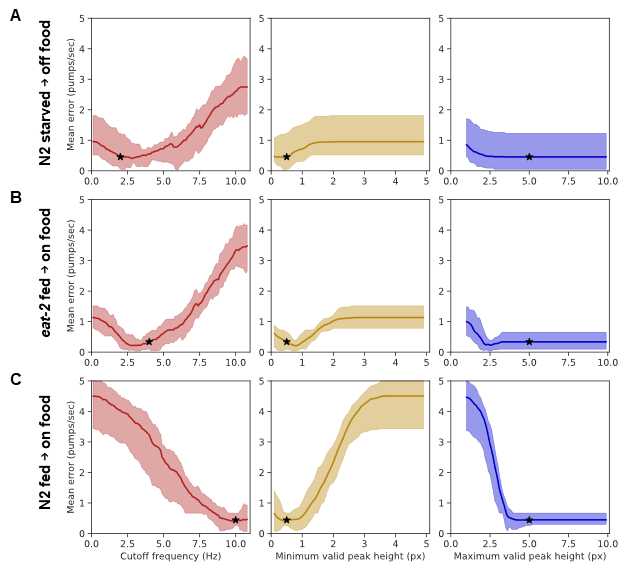

Supplement: S4 Fig — A parameter sweep was conducted for three of the eight experimental conditions discussed in the text, each with a different pumping rate: starved N2 worms off food (A), fed eat-2 worms on food (B), and fed N2 worms on food (C). The results for each sweep were calculated as the mean error when compared to two expert manual labelers, where the mean across all samples (N = 5) is represented by a line and the min-max range is represented by a shaded region. Star denotes the parameter value used for each condition in this study. (TIF) [file pcbi.1014489.s004.tif]

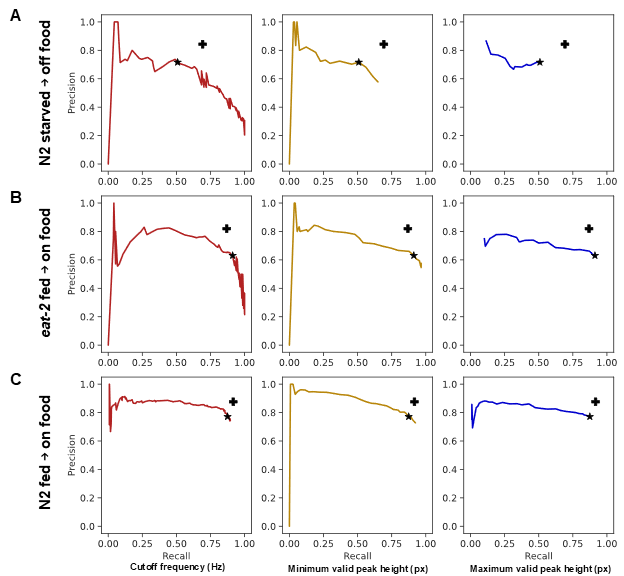

Supplement: S5 Fig — A precision-recall curve was provided for each parameter sweep for three of the eight experimental conditions discussed in the text, each with a different pumping rate: starved N2 worms off food (A), fed eat-2 worms on food (B), and fed N2 worms on food (C). PumpKin’s estimated pump times were matched 1:1 to a consensus between the two expert manual labelers’ reported pump times within a time window Δt (A: Δt = 1.2, B: Δt = 0.7, Δt = 0.2). The final precision and recall values were calculated on the pooled counts across the samples in each experimental condition (N = 5). Star denotes the parameter value used for each condition in this study. Plus sign (+) indicates the labeler-labeler precision/recall. (TIF) [file pcbi.1014489.s005.tif]
