# Supplementary material for: PumpKin: A machine-learning pipeline for automatically tracking localized kinematics in freely moving C. elegans
Source: PLoS Comput Biol. 2026 Jul 17;22(7):e1014489. doi: 10.1371/journal.pcbi.1014489 (PMC13399524; doi:10.1371/journal.pcbi.1014489)
Supplement: Attachment — Submitted filename: PumpKin_ResponseLetter.pdf [file pcbi.1014489.s021.pdf]

We thank the reviewers for a thorough read and for their helpful critiques. We have responded to all their questions and comments here and in the manuscript text. We believe the changes suggested by the reviewers have clarified and strengthened the arguments presented in this manuscript. Below are our point-by-point responses to each reviewer's comments.

Reviewer: 1

Recommendation: The manuscript presents a computational pipeline, named PumpKin, to track pumping rate in *C. elegans*. The work could be a potentially useful tool for *C. elegans* studies. However, I have the following comments regarding the manuscript:

1. The title, abstract, introduction, and discussion prominently claim generalizations of PumpKin to "freely moving organisms" broadly, yet the entire validation is performed on a single organism, a single behavior, and a single imaging modality. The claim of generalization is therefore speculative. I would recommend the authors either: (a) demonstrate PumpKin on at least one additional organism/behavior to substantiate the applicability to "freely moving organisms," or (b) revise their language throughout the manuscript.

We agree with the reviewer that the paper does not provide evidence to support PumpKin's ability to generalize to behaviors in other organisms. To address this issue, we have followed the reviewer's recommendation to revise the language throughout the manuscript to specify PumpKin's application to *C. elegans*, and we have moved any discussion of generalization to the Discussion as a potential for future work (page 12, final paragraph).

2. The Butterworth filter cutoff frequency is derived from the expected pumping rate of the strain, meaning we must already have a reasonable estimate of the behavior we are trying to measure. The authors briefly acknowledge that the lawn-entry experiment uses separate parameters for pre- and post-lawn entry. However, it remains unclear how sensitive the results are to a different choice of cutoff frequency, and what happens in novel conditions where the expected rate is unknown. A parameter sensitivity analysis is important here and should be included.

We wholeheartedly agree that a parameter sensitivity analysis is important for assessing how precise the user's parameter selection must be when estimating the pumping rate in novel data. We would like to emphasize here that, in the case of *C. elegans* research, users should have a general estimate for the cutoff frequency as it is based off of the average expected pumping rate for the strain of interest. As suggested, we have conducted a parameter sweep for the three parameters that the user must tune for novel data: the cutoff frequency (Hz) for the low-pass Butterworth filter and the minimum and maximum peak heights (in pixels) for the biphasic peak detector. The inverse of the cutoff frequency (seconds) is used as the peak distance threshold for the biphasic peak detector, so we only include the cutoff frequency to avoid redundancy.

We completed three separate sweeps using three conditions from our validation set that cover three different expected pumping rates: N2 starved -> off food (lowest, 0.5-1 Hz), *eat-2* fed -> on food (middle, 1-2 Hz), and N2 fed -> on food (highest, 4-5 Hz). For each sweep, the mean error was calculated between PumpKin's estimate and the estimates of two manual labelers. We provide the results from these parameter sweeps in Supplemental Figure 4 of our revised document, and we denote the parameter value used for each condition with a star.

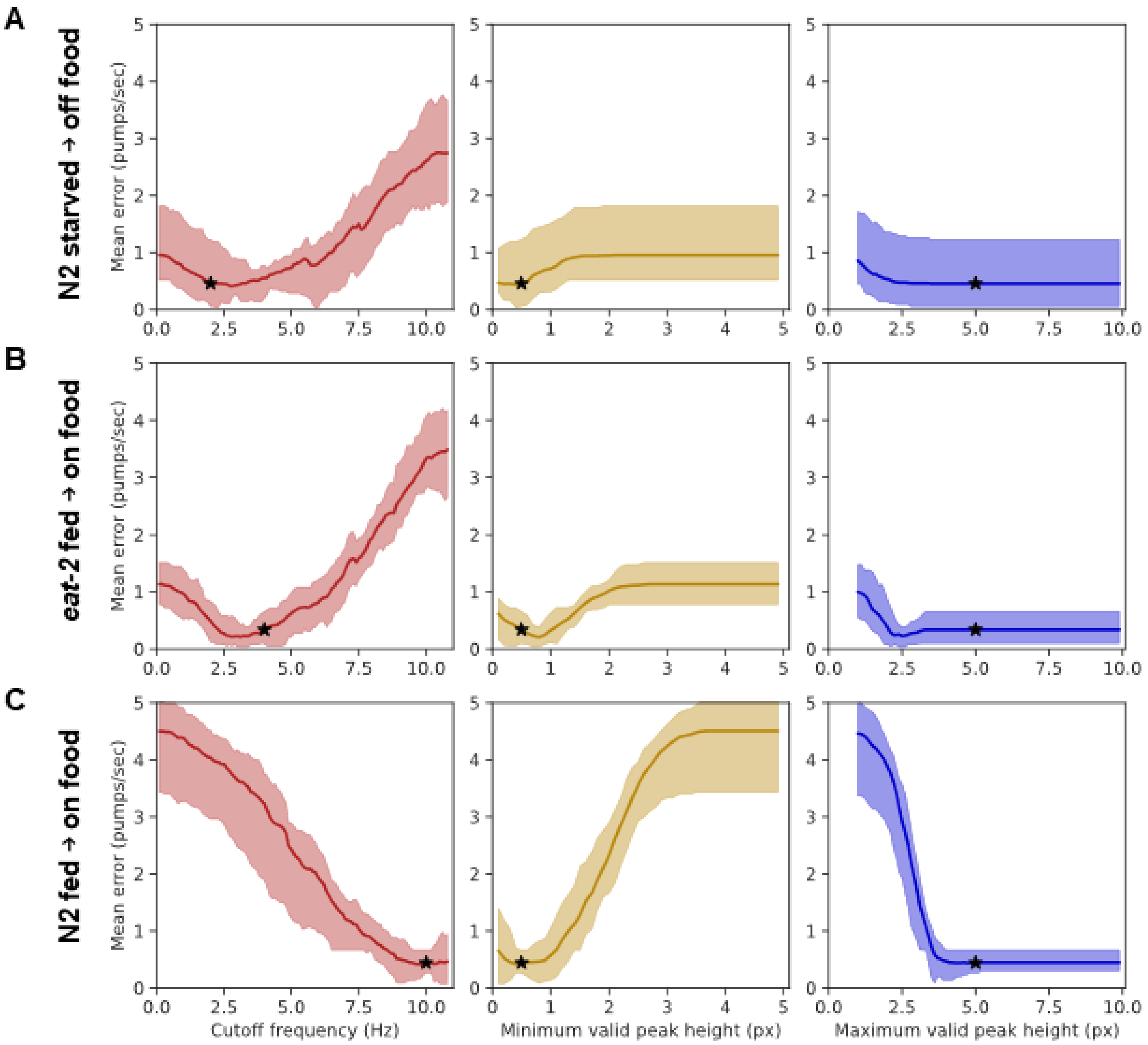

Supplemental Figure 4: A parameter sweep for PumpKin's cutoff frequency, minimum valid peak height, and maximum valid peak height across three experimental conditions. A parameter sweep was conducted for three of the eight experimental conditions discussed in the text, each with a different pumping rate: starved N2 worms off food (A), fed *eat-2* worms on food (B), and fed N2 worms on food (C). The results for each sweep were calculated as the mean error when compared to two expert manual labelers, where the mean across all samples (N=5) is represented by a line and the min-max range is represented by a shaded region. Star denotes the parameter value used for each condition in this study.

3. Table 1 compares PumpKin against prior methods, but no quantitative comparison is made against any of them. PharaGlow in particular is directly relevant, as it also tracks pharyngeal pumping in freely moving worms. The claim that PumpKin represents an advance cannot be fully evaluated without benchmarking. If running PharaGlow on the same dataset is infeasible, this limitation should be explicitly stated and justified.

We would like to clarify that existing techniques, like PharaGlow, require specialized imaging setups and genetic tools that were infeasible for the scope of this work. For example, PharaGlow depends on pharyngeal fluorescent reporters, which requires the user to construct a fluorescent transgenic strain for each genotype of interest. This time burden, coupled with the overhead associated with their epifluorescence-based imaging pipeline made a comparison against PharaGlow impractical. It is exactly one of the reasons we developed our technique.

We strongly agree, however, with the reviewer's recommendation that this infeasibility of benchmarking against PharaGlow should be stated and justified in the text. We have added a statement in the Discussion on our justification for limiting our comparisons to manual estimation, which remains the primary method for measuring pumping in this field despite the development of automated techniques (page 12, lines 250-259).

4. The reported R^2 values of 0.99 and 0.97 in Fig. 3B are not informative and arguably misleading given the structure of the underlying data. The condition-averaged pumping rates form two tight, well-separated clusters, with eat-2 conditions near 1–1.5 Hz and N2 conditions near 4–5 Hz. In this bimodal structure, a high R^2 is mathematically guaranteed by the large between-cluster separation (~3–4 Hz), which completely dominates the variance calculation regardless of how accurately PumpKin performs within each cluster.

We thank the reviewer for pointing out that our reported R^2 values may be misleading due to the large difference between the lower and higher pumping rates included in our dataset. We have plotted the lower (<3 Hz) and higher (>3 Hz) pumping rates separately to resolve this in the updated Figure 3. We also include all data points for each condition rather than only plotting the condition averages to provide a clearer visual of the variance within the data. We report the separate Pearson's R values for the comparison to each of our two labelers in a new Supplemental Table, and we have updated Figure 3B and Supplemental Figure 1B (which compares manual labelers' estimates to one another in a similar manner) accordingly. The Pearson's R values for Supplemental Figure 1B are provided in a separate Supplemental Table.

--

Updated Figure 3 + associated Supplemental Table containing Pearson's R values for Panel B:

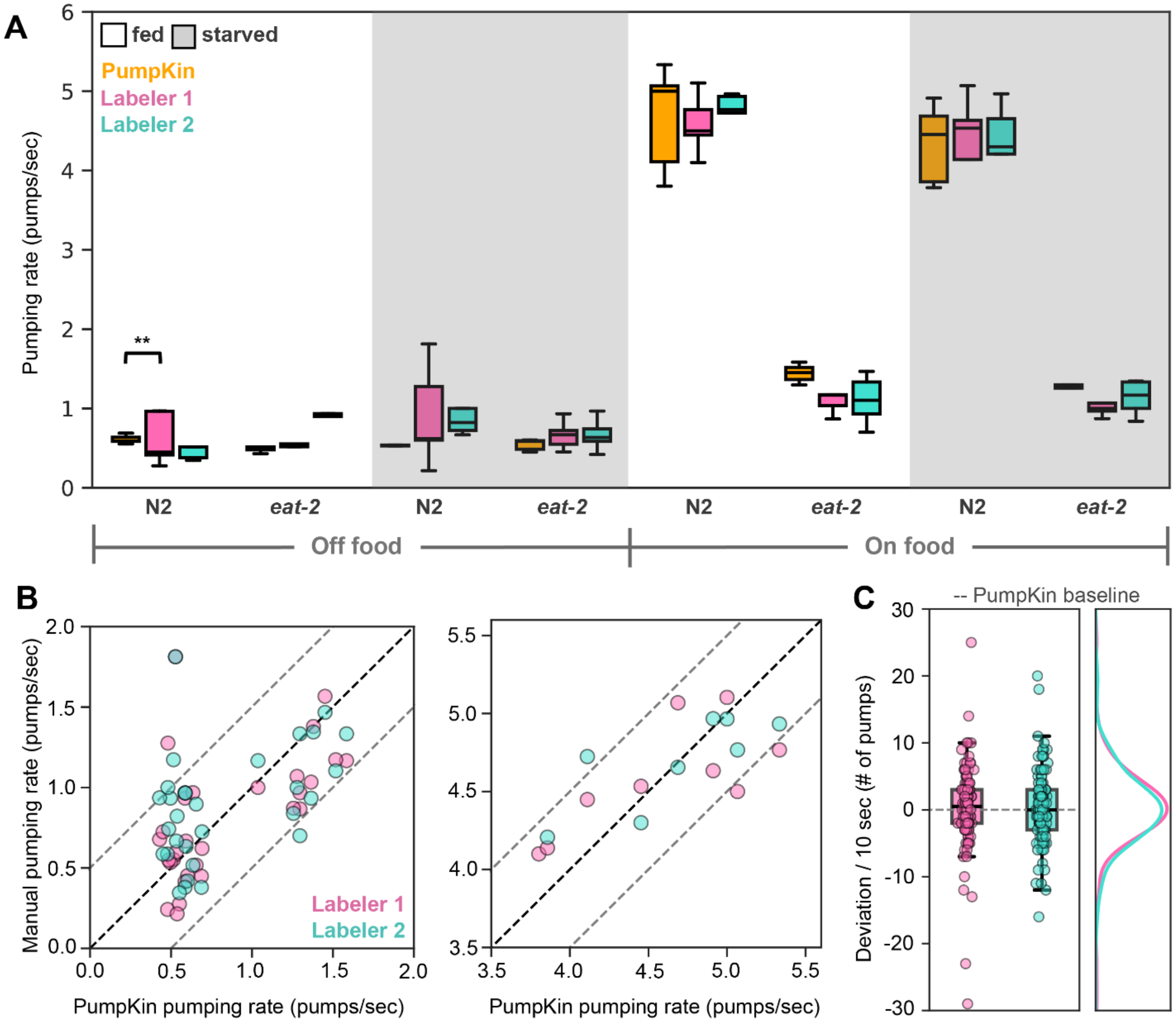

Fig 3.

PumpKin generates pumping rate estimates consistent with those obtained by expert manual labelers and that capture known differences in pumping due to satiety and genetic mutation. (A) Average pumping rates estimated by PumpKin are compared with those obtained by two manual labelers of high expertise. N=5 per box. Statistical significance was determined via unpaired t-test: *$p < 0.05$, **$p < 0.01$, ***$p < 0.001$ (B) Comparison of the average pumping rates estimated by PumpKin and two expert labelers, separated by low (0-2 pumps/sec) and high (3.5-5.5 pumps/sec) pumping rates. The center dashed line represents perfect correlation, and the additional dashed lines represent perfect correlation shifted by ±0.5 pumps/sec. (C) Deviation in pump count between PumpKin and expert estimates within a 10-second clip. 30-second videos were divided into (3) 10-second video clips and pump count discrepancies were calculated by subtracting the PumpKin counts from the expert counts. N=120 per bar. Dashed line represents PumpKin baseline. KDE distributions for each labeler are shown on the right.

Supplemental Table 3. All Pearson's R values from comparing PumpKin's average pumping rates to those obtained by two expert labelers.

Pumping rates (pumps/sec)	Labeler 1	Labeler 2
**0 - 3**	0.54	0.45
**3 - 6**	0.70	0.80

--

Updated Supplemental Figure 1 and + associated Supplemental Table containing Pearson's R values for Panel B.

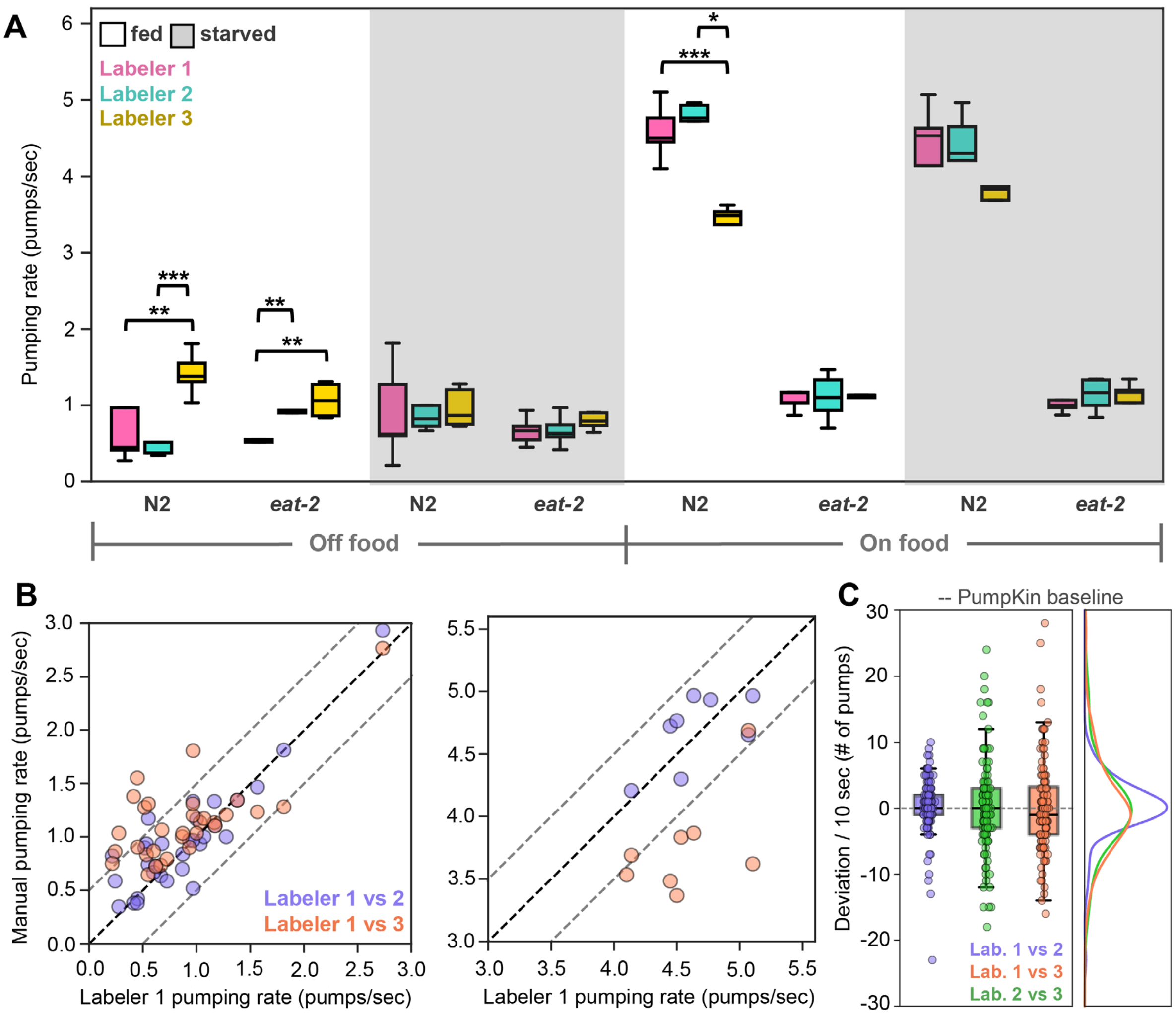

Supplemental Figure 1: Manual estimates of pumping rate are dependent on experience. (A) Average pumping rates obtained by three double-blinded manual labelers of varying expertise. Labelers are categorized by pump counting expertise, with Labeler 1 having the most experience and Labeler 3 having the least. N=5 per box. Statistical significance was determined via unpaired t-test: *$p < 0.05$, **$p < 0.01$, ***$p < 0.001$. (B) Comparison of the condition-averaged pumping rates of Labelers 2 and 3 with those of Labeler 1, separated by low (0-3 pumps/sec) and high (3-6 pumps/sec) pumping rates. The center dashed line represents perfect correlation, and the additional dashed lines represent perfect correlation shifted by ±0.5 Hz. (C) Deviation in pump count between manual estimates within a 10-second clip. 30-second videos were divided into (3) 10-second video clips and pump count discrepancies were calculated by subtracting the PumpKin counts from the manual counts. N=120 per bar. KDE distributions for each labeler pairing are shown on the right.

Supplemental Table 1. All Pearson's R values from comparing Labeler 1's average pumping rates to those obtained by two additional labelers.

Pumping rates (pumps/sec)	Labeler 2	Labeler 3
**0 - 3**	0.79	0.36
**3 - 6**	0.79	0.52

5. Using only 5 worms per condition for the primary validation comparison limits the statistical power to detect differences between PumpKin and human annotators. This should be acknowledged as a limitation of the study.

We agree with the reviewer that the low N number per condition in our validation set is a valid limitation of this work. We have followed the reviewer's recommendation and stated this as a limitation in the Discussion (page 12, lines 247-250), while also clarifying that human annotation time is the bottleneck in obtaining a higher N.

6. The peak detector's sensitivity and specificity should be characterized, ideally as a function of the key tunable parameters. At present, no evaluation of how parameter choices affect false positive or false negative rates is provided.

We fully agree with the reviewer that, in addition to a parameter sweep, we should also characterize the peak detector's ability to label individual pumps accurately. To fill this gap, we have calculated and plotted the precision-recall curves for the peak detector across the same three conditions (N2 starved -> off food, *eat-2* fed -> on food, and N2 fed -> on food) and same three parameters (cutoff frequency, minimum peak height, and maximum peak height) used for the parameter sweep we provided in response to this reviewer's Comment #2.

We would like to specify that, in this context, there is no true "ground truth." Thus, we compared PumpKin's labeled pump times to a consensus labeling of our two manual labelers. Consensus pump times were defined as pumps identified by both labelers within a ±Δt window, where Δt was selected based on the expected pumping rate of the sample. Pumps were matched on a 1:1 basis to prevent repeated matches. The final consensus time was calculated as the midpoint of the two matched pump times. PumpKin's pump times were subsequently matched with the consensus pump times using the same ±Δt window and 1:1 matching procedure. We provide the specific Δt values used for each condition below and in the caption for Supplemental Figure 5:

Experimental condition	Expected pumping rate (pumps/sec)	Δt (seconds)
**N2 starved -> no food**	0.5 - 1	1.2
***eat-2* fed -> food**	1 - 2	0.7
**N2 fed -> food**	4 - 5	0.2

In the context of pumping, we define a true positive as a pump event labeled by both PumpKin and the consensus, a false positive as a pump event only labeled by PumpKin, and a false negative as a pump event only labeled by the consensus. Since N=5 samples per experimental condition, the final precision and recall were calculated on the pooled counts across samples as follows:

$$precision = \frac{\Sigma TP}{\Sigma TP + \Sigma FP}$$

$$recall = \frac{\Sigma TP}{\Sigma TP + \Sigma FN}$$

We have added the results from this precision-recall analysis to Supplemental Figure 5, and denote the parameter value used for each condition with a star and compare this to the labeler-labeler precision/recall, which is denoted by a plus (+). We would like to emphasize that the labeler-labeler precision/recall represents how well labelers agree with each other when labeling exact peak times, and it is not representative of them performing better (or worse) than PumpKin since labeler estimates are not a perfect "ground truth."

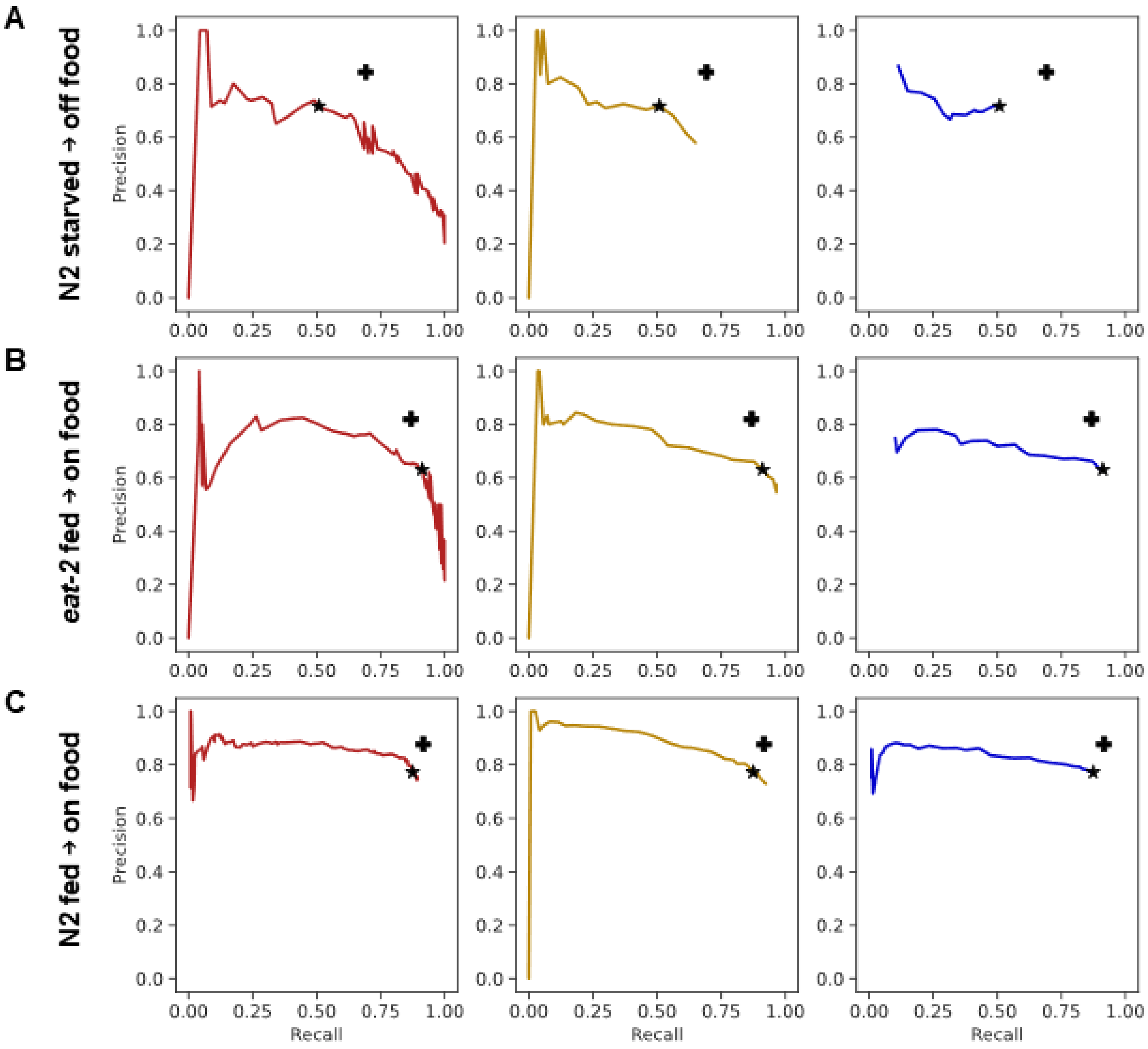

Supplemental Figure 5: Precision-recall analysis for PumpKin's biphasic peak detector based on parameter sweeps over the cutoff frequency, minimum valid peak height, and maximum valid peak height across three experimental conditions. A precision-recall curve was provided for each parameter sweep for three of the eight experimental conditions discussed in the text, each with a different pumping rate: starved N2 worms off food (A), fed *eat-2* worms on food (B), and fed N2 worms on food (C). PumpKin's estimated pump times were matched 1:1 to a consensus between the two expert manual labelers' reported pump times within a time window Δt (A: Δt = 1.2, B: Δt = 0.7, Δt = 0.2). The final precision and recall values were calculated on the pooled counts across the samples in each experimental condition (N=5). Star denotes the parameter value used for each condition in this study. Plus sign (+) indicates the labeler-labeler precision/recall.

Reviewer: 2

Recommendation: This manuscript introduces PumpKin, a machine-learning pipeline designed to quantify localized, single-structure kinematics in freely moving animals. The authors combine sequential Faster R-CNN detection, motion compensation, sparse optical flow, Butterworth filtering, and biphasic peak detection to track *C. elegans* pharyngeal pumping without fixation or fluorescent labeling. Validation across eight experimental conditions (genotype, satiety, and food presence) demonstrates high agreement with expert manual annotations for both average pumping rate ($R^2 \geq 0.97$) and instantaneous dynamics. The authors further use the pipeline to uncover a shared basal off-food pumping rate (~0.5 Hz) across strains and satiety states. While this work addresses a meaningful technical gap, I have several concerns,

1. The authors evaluate only the Faster R-CNN model, although other state-of-the-art detection architectures such as YOLO and Real-Time Detection Transformer (RT-DETR) are available. It would strengthen the manuscript to include comparisons with these alternative detectors and report their performance relative to the chosen baseline. Such benchmarking would help clarify whether the reported improvements are specific to Faster R-CNN or generalizable across detection frameworks.

We agree that alternative object detectors, such as YOLO and RT-DETR, could be used in place of Faster R-CNN when using PumpKin. However, PumpKin only operates on pixel-level information within the detected grinder and is insensitive to small optimizations (e.g., tighter bounding boxes) in detections; therefore, its performance is not specific to Faster R-CNN and instead generalizes to any detector that provides consistent object detection. Furthermore, benchmarking against other detection architectures would require extensive tuning of each architecture. We therefore focus on demonstrating the performance of PumpKin independent of detector choice.

2. Although the manuscript describes several tunable components (e.g., Butterworth cutoff frequency, peak distance threshold, and peak height threshold; Methods, pp. 14–15), the sensitivity of the method to these parameters remains unclear. Specifically: 1) How sensitive is performance to variations in these parameters? 2) Were the parameters fixed across all experimental conditions? 3) How much user intervention is required when adapting the pipeline to a new organism, behavior, or different imaging conditions?

We agree that the sensitivity of PumpKin's results to its parameters should be characterized, and we address the reviewer's specific questions below:

1) ***How sensitive is performance to variations in these parameters?***
   In response to the previous reviewer's comment #2, we have added a parameter sweep over the cutoff frequency and the minimum and maximum peak heights. We clarified in our response to reviewer #1 that the peak distance threshold is determined using the same cutoff

frequency used for the Butterworth filter, so we only include a sweep for the cutoff frequency to avoid redundancy. We've copied the results of this sweep below, and have added it to the text as Supplemental Figure 4:

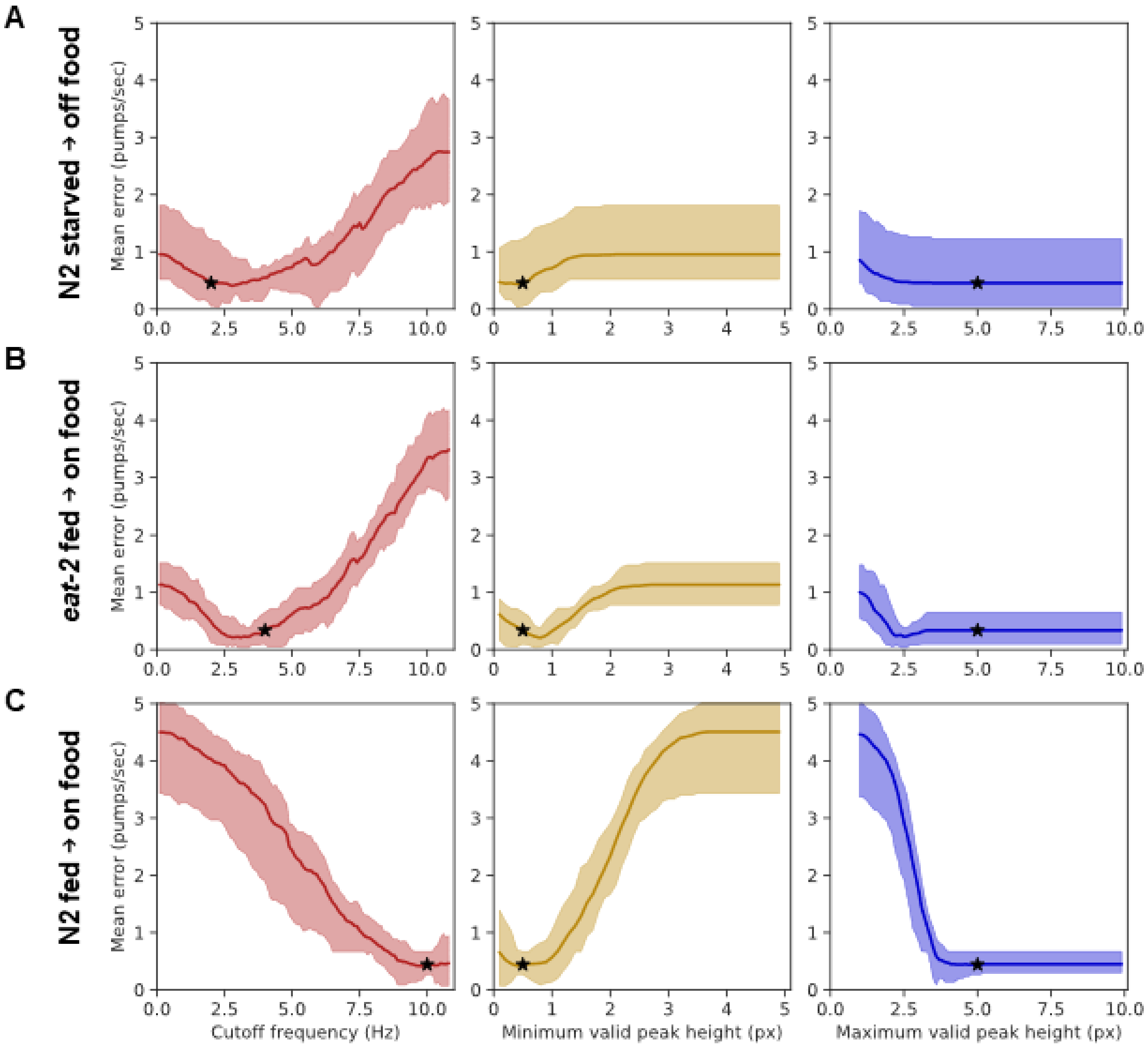

Supplemental Figure 4: A parameter sweep for PumpKin's cutoff frequency, minimum valid peak height, and maximum valid peak height across three experimental conditions. A parameter sweep was conducted for three of the eight experimental conditions discussed in the text, each with a different pumping rate: starved N2 worms off food (A), fed *eat-2* worms on food (B), and fed N2 worms on food (C). The results for each sweep were calculated as the mean error when compared to two expert manual labelers, where the mean across all samples (N=5) is represented by a line and the min-max range is represented by a shaded region. Star denotes the parameter value used for each condition in this study.

2) Were the parameters fixed across all experimental conditions?

We fixed the minimum and maximum peak heights for the biphasic peak detector across all experimental conditions, but we select the cutoff frequency based on the expected pumping rate of the worms in each condition. We have summarized these in the table below, and in Supplemental Table 2:

Supplemental Table 2. All PumpKin parameter values used for each experimental condition.

Experimental condition	Cutoff frequency (Hz)	Min peak height (px)	Max peak height (px)
N2 fed -> no food	1	0.5	5
eat-2 fed -> no food	1	0.5	5
N2 starved -> no food	1	0.5	5
eat-2 starved -> no food	1	0.5	5
N2 fed -> food	10	0.5	5
eat-2 fed -> food	2	0.5	5
N2 starved -> food	10	0.5	5
eat-2 starved -> food	2	0.5	5

3) ***How much user intervention is required when adapting the pipeline to a new organism, behavior, or different imaging conditions?***
The three parameters that should be adjusted by the user when recording a new organism/behavior or when using a new imaging system are the cutoff frequency and the minimum and maximum peak heights. The cutoff frequency should be determined empirically based on the rate of the behavior (measured in events/second), and the min/max peak heights should be determined via parameter sweep for the best results. We thank the reviewer for this question and have added these comments to the text.

3. The manuscript states that training frames were selected using DeepLabCut k-means clustering and that models were fine-tuned for 50 epochs (Methods, p. 13). However, important training details are missing, including: the number of annotated frames used per model, the train/validation/test split strategy, whether evaluation videos were fully held out from the training dataset.

We thank the reviewer for pointing out that these details were not explicitly stated in the text. While we originally referred to our original work that summarizes the application of Faster R-CNN to tracking the pharyngeal bulb and grinder (EZ-FRCNN: A Fast, Accessible and Robust Deep Learning Package for Object Detection Applications from Ethology to Cell Biology | bioRxiv), we have replaced this reference with the details of model training, including the number of frames, train/test split, and clarifying that evaluation videos were fully held out in validating each Faster R-CNN model.

4. In Figure 4, the number of manual human annotations appears limited. It is unclear why the uncertainty (grey shaded region) is very small in some cases but much larger in others. Since the model's reported performance is evaluated relative to this uncertainty band, the variability across conditions requires further explanation. Additionally, in panel D, there appears to be a grey uncertainty region despite only one manual estimate being shown. The authors should clarify how this uncertainty was computed in that case.

We thank the reviewer for pointing out a lack of clarity in the calculation of the confidence intervals (i.e., gray shaded regions) of Figure 4, panels A and D. We followed the reviewer's recommendation and have specified in both the main text (page 8, lines 166-167 and page 9, lines 188-189) and figure caption that the confidence intervals in Figure 4A are calculated based on a 1.5 pooled standard deviation across three expert manual labelers, and the confidence interval in Figure 4D is calculated based on a flat ±15% tolerance for a single expert manual labeler.

5. The introduction appropriately discusses the limitations of single-keypoint pose tracking due to drift. However, the manuscript would be strengthened by including direct benchmarking against pose estimation tools such as DeepLabCut or SLEAP trained specifically for grinder localization. The authors should make more quantitative comparisons with peer models as listed in Table 1 to deminstrate its advances.

We thank the reviewer for their recommendation, and we have added a direct reference to Figure Supplement 1 of our previous work (EZ-FRCNN: A Fast, Accessible and Robust Deep Learning Package for Object Detection Applications from Ethology to Cell Biology | bioRxiv) in the text (page 3, lines 46-48), where we benchmarked Faster R-CNN's performance on grinder tracking against DeepLabCut and SLEAP.

We wholeheartedly agree with the reviewer that benchmarking against existing techniques, such as those listed in Table 1, would make this work stronger. Following Reviewer #1's recommendation, we have added more detailed reasoning for why we cannot benchmark against these methods (they use specialized setups/techniques we do not have access to, and/or they are too restrictive to the worm to be considered accurate). We also provide a visual example of why thresholding (a technique many of these methods use) is ineffective in tracking the grinder in Supplemental Figure 1 of this manuscript.
