# Supplementary material for: PumpKin: A machine-learning pipeline for automatically tracking localized kinematics in freely moving C. elegans
Source: PLoS Comput Biol. 2026 Jul 17;22(7):e1014489. doi: 10.1371/journal.pcbi.1014489 (PMC13399524; doi:10.1371/journal.pcbi.1014489)
Supplement: Peer review history

# Peer review: Decision Letter 0

**DOI:** 10.1371/journal.pcbi.1014489.r001

**Authors:** Feng Fu, Thomas Zdyrski

31 Mar 2026

PCOMPBIOL-D-26-00134

PumpKin: A machine-learning pipeline for automatically tracking localized kinematics in freely moving organisms

PLOS Computational Biology

Dear Dr. Lu,

Thank you for submitting your manuscript to PLOS Computational Biology. After careful consideration, we feel that it has merit but does not fully meet PLOS Computational Biology's publication criteria as it currently stands. Therefore, we invite you to submit a revised version of the manuscript that addresses the points raised during the review process.

Please submit your revised manuscript by May 31 2026 11:59PM. If you will need more time than this to complete your revisions, please reply to this message or contact the journal office at ploscompbiol@plos.org. When you're ready to submit your revision, log on to https://www.editorialmanager.com/pcompbiol/ and select the 'Submissions Needing Revision' folder to locate your manuscript file.

Please include the following items when submitting your revised manuscript:

* A letter that responds to each point raised by the editor and reviewer(s). You should upload this letter as a separate file labeled 'Response to Reviewers'. This file does not need to include responses to formatting updates and technical items listed in the 'Journal Requirements' section below.

* A marked-up copy of your manuscript that highlights changes made to the original version. You should upload this as a separate file labeled 'Revised Manuscript with Track Changes'.

* An unmarked version of your revised paper without tracked changes. You should upload this as a separate file labeled 'Manuscript'.

If you would like to make changes to your financial disclosure, competing interests statement, or data availability statement, please make these updates within the submission form at the time of resubmission. Guidelines for resubmitting your figure files are available below the reviewer comments at the end of this letter

We look forward to receiving your revised manuscript.

Kind regards,

Thomas Zdyrski

Guest Editor

PLOS Computational Biology

Feng Fu

Section Editor

PLOS Computational Biology

**Additional Editor Comments :**

Dear Authors,

We recommend a major revision incorporating the reviewers' suggestions. Please send a revised copy after updating the manuscript.

Thank you kindly

**Journal Requirements:**

1) Please ensure that the CRediT author contributions listed for every co-author are completed accurately and in full.

At this stage, the following Authors/Authors require contributions: Erin Shappell, Debra Buggs, Jennah Walcott, and Hang Lu. Please ensure that the full contributions of each author are acknowledged in the "Add/Edit/Remove Authors" section of our submission form.

The list of CRediT author contributions may be found here: https://journals.plos.org/ploscompbiol/s/authorship#loc-author-contributions

2) We ask that a manuscript source file is provided at Revision. Please upload your manuscript file as a .doc, .docx, .rtf or .tex. If you are providing a .tex file, please upload it under the item type u2018LaTeX Source Fileu2019 and leave your .pdf version as the item type u2018Manuscriptu2019.

3) Please upload all main figures as separate Figure files in .tif or .eps format. For more information about how to convert and format your figure files please see our guidelines:

https://journals.plos.org/ploscompbiol/s/figures

4) Please amend your detailed Financial Disclosure statement. This is published with the article. It must therefore be completed in full sentences and contain the exact wording you wish to be published.

1) State what role the funders took in the study. If the funders had no role in your study, please state: “The funders had no role in study design, data collection and analysis, decision to publish, or preparation of the manuscript.”

5) Some material included in your submission may be copyrighted. According to PLOS’s copyright policy, authors who use figures or other material (e.g., graphics, clipart, maps) from another author or copyright holder must demonstrate or obtain permission to publish this material under the Creative Commons Attribution 4.0 International (CC BY 4.0) License used by PLOS journals. Please closely review the details of PLOS’s copyright requirements here: PLOS Licenses and Copyright. If you need to request permissions from a copyright holder, you may use PLOS's Copyright Content Permission form.

Please respond directly to this email and provide any known details concerning your material's license terms and permissions required for reuse, even if you have not yet obtained copyright permissions or are unsure of your material's copyright compatibility. Once you have responded and addressed all other outstanding technical requirements, you may resubmit your manuscript within Editorial Manager.

Potential Copyright Issues:

Figures 1, 2, 4c, and supplementary videos. Please confirm (a) that you are the photographer; or (b) provide written permission from the photographer to publish the photo(s) under our CC BY 4.0 license.

Note: If the reviewer comments include a recommendation to cite specific previously published works, please review and evaluate these publications to determine whether they are relevant and should be cited. There is no requirement to cite these works unless the editor has indicated otherwise.

**Reviewers' comments:**

Reviewer's Responses to Questions

Reviewer #1: The manuscript presents a computational pipeline, named PumpKin, to track pumping rate in C. elegans. The work could be a potentially useful tool for C. elegans studies. However, I have the following comments regarding the manuscript:

1. The title, abstract, introduction, and discussion prominently claim generalizations of PumpKin to "freely moving organisms" broadly, yet the entire validation is performed on a single organism, a single behavior, and a single imaging modality. The claim of generalization is therefore speculative. I would recommend the authors either: (a) demonstrate PumpKin on at least one additional organism/behavior to substantiate the applicability to "freely moving organisms," or (b) revise their language throughout the manuscript.

2. The Butterworth filter cutoff frequency is derived from the expected pumping rate of the strain, meaning we must already have a reasonable estimate of the behavior we are trying to measure. The authors briefly acknowledge that the lawn-entry experiment uses separate parameters for pre- and post-lawn entry. However, it remains unclear how sensitive the results are to a different choice of cutoff frequency, and what happens in novel conditions where the expected rate is unknown. A parameter sensitivity analysis is important here and should be included.

3. Table 1 compares PumpKin against prior methods, but no quantitative comparison is made against any of them. PharaGlow in particular is directly relevant, as it also tracks pharyngeal pumping in freely moving worms. The claim that PumpKin represents an advance cannot be fully evaluated without benchmarking. If running PharaGlow on the same dataset is infeasible, this limitation should be explicitly stated and justified.

4. The reported R² values of 0.99 and 0.97 in Fig. 3B are not informative and arguably misleading given the structure of the underlying data. The condition-averaged pumping rates form two tight, well-separated clusters, with eat-2 conditions near 1–1.5 Hz and N2 conditions near 4–5 Hz. In this bimodal structure, a high R² is mathematically guaranteed by the large between-cluster separation (~3–4 Hz), which completely dominates the variance calculation regardless of how accurately PumpKin performs within each cluster.

5. Using only 5 worms per condition for the primary validation comparison limits the statistical power to detect differences between PumpKin and human annotators. This should be acknowledged as a limitation of the study.

6. The peak detector's sensitivity and specificity should be characterized, ideally as a function of the key tunable parameters. At present, no evaluation of how parameter choices affect false positive or false negative rates is provided.

Reviewer #2: This manuscript introduces PumpKin, a machine-learning pipeline designed to quantify localized, single-structure kinematics in freely moving animals. The authors combine sequential Faster R-CNN detection, motion compensation, sparse optical flow, Butterworth filtering, and biphasic peak detection to track C. elegans pharyngeal pumping without fixation or fluorescent labeling. Validation across eight experimental conditions (genotype, satiety, and food presence) demonstrates high agreement with expert manual annotations for both average pumping rate (R² ≥ 0.97) and instantaneous dynamics. The authors further use the pipeline to uncover a shared basal off-food pumping rate (~0.5 Hz) across strains and satiety states. While this work addresses a meaningful technical gap, I have several concerns,

1. The authors evaluate only the Faster R-CNN model, although other state-of-the-art detection architectures such as YOLO and Real-Time Detection Transformer (RT-DETR) are available. It would strengthen the manuscript to include comparisons with these alternative detectors and report their performance relative to the chosen baseline. Such benchmarking would help clarify whether the reported improvements are specific to Faster R-CNN or generalizable across detection frameworks.

2. Although the manuscript describes several tunable components (e.g., Butterworth cutoff frequency, peak distance threshold, and peak height threshold; Methods, pp. 14–15), the sensitivity of the method to these parameters remains unclear. Specifically: 1) How sensitive is performance to variations in these parameters? 2) Were the parameters fixed across all experimental conditions? 3) How much user intervention is required when adapting the pipeline to a new organism, behavior, or different imaging conditions?

3. The manuscript states that training frames were selected using DeepLabCut k-means clustering and that models were fine-tuned for 50 epochs (Methods, p. 13). However, important training details are missing, including: the number of annotated frames used per model, the train/validation/test split strategy, whether evaluation videos were fully held out from the training dataset.

4. In Figure 4, the number of manual human annotations appears limited. It is unclear why the uncertainty (grey shaded region) is very small in some cases but much larger in others. Since the model’s reported performance is evaluated relative to this uncertainty band, the variability across conditions requires further explanation. Additionally, in panel D, there appears to be a grey uncertainty region despite only one manual estimate being shown. The authors should clarify how this uncertainty was computed in that case.

5. The introduction appropriately discusses the limitations of single-keypoint pose tracking due to drift. However, the manuscript would be strengthened by including direct benchmarking against pose estimation tools such as DeepLabCut or SLEAP trained specifically for grinder localization. The authors should make more quantitative comparisons with peer models as listed in Table 1 to deminstrate its advances.

**********

**Have the authors made all data and (if applicable) computational code underlying the findings in their manuscript fully available?**

The PLOS Data policy requires authors to make all data and code underlying the findings described in their manuscript fully available without restriction, with rare exception (please refer to the Data Availability Statement in the manuscript PDF file). The data and code should be provided as part of the manuscript or its supporting information, or deposited to a public repository. For example, in addition to summary statistics, the data points behind means, medians and variance measures should be available. If there are restrictions on publicly sharing data or code —e.g. participant privacy or use of data from a third party—those must be specified.

Reviewer #1: None

Reviewer #2: Yes

**********

PLOS authors have the option to publish the peer review history of their article (what does this mean?). If published, this will include your full peer review and any attached files.

If you choose “no”, your identity will remain anonymous but your review may still be made public.

**Do you want your identity to be public for this peer review?**  For information about this choice, including consent withdrawal, please see our Privacy Policy.

Reviewer #1: No

Reviewer #2: No

[NOTE: If reviewer comments were submitted as an attachment file, they will be attached to this email and accessible via the submission site. Please log into your account, locate the manuscript record, and check for the action link "View Attachments". If this link does not appear, there are no attachment files.]

**Figure resubmission:**

While revising your submission, we strongly recommend that you use PLOS’s NAAS tool (https://ngplosjournals.pagemajik.ai/artanalysis) to test your figure files. NAAS can convert your figure files to the TIFF file type and meet basic requirements (such as print size, resolution), or provide you with a report on issues that do not meet our requirements and that NAAS cannot fix.

After uploading your figures to PLOS’s NAAS tool - https://ngplosjournals.pagemajik.ai/artanalysis, NAAS will process the files provided and display the results in the "Uploaded Files" section of the page as the processing is complete. If the uploaded figures meet our requirements (or NAAS is able to fix the files to meet our requirements), the figure will be marked as "fixed" above. If NAAS is unable to fix the files, a red "failed" label will appear above. When NAAS has confirmed that the figure files meet our requirements, please download the file via the download option, and include these NAAS processed figure files when submitting your revised manuscript.

**Reproducibility:**

To enhance the reproducibility of your results, we recommend that authors of applicable studies deposit laboratory protocols in protocols.io, where a protocol can be assigned its own identifier (DOI) such that it can be cited independently in the future. Additionally, PLOS ONE offers an option to publish peer-reviewed clinical study protocols. Read more information on sharing protocols at https://plos.org/protocols?utm_medium=editorial-email&utm_source=authorletters&utm_campaign=protocols

# Author comment: Author response to Decision Letter 1

**DOI:** 10.1371/journal.pcbi.1014489.r002

28 May 2026

Attachment Submitted filename: PumpKin_ResponseLetter.pdf

# Editor report: Decision Letter 1

**DOI:** 10.1371/journal.pcbi.1014489.r003

**Authors:** Feng Fu, Thomas Zdyrski

15 Jun 2026

PCOMPBIOL-D-26-00134R1

PumpKin: A machine-learning pipeline for automatically tracking localized kinematics in freely moving C. elegans

PLOS Computational Biology

Dear Dr. Lu,

Thank you for submitting your manuscript to PLOS Computational Biology. After careful consideration, we feel that it has merit but does not fully meet PLOS Computational Biology's publication criteria as it currently stands. Therefore, we invite you to submit a revised version of the manuscript that addresses the points raised during the review process.

Please submit your revised manuscript by Aug 15 2026 11:59PM. If you will need more time than this to complete your revisions, please reply to this message or contact the journal office at ploscompbiol@plos.org. When you're ready to submit your revision, log on to https://www.editorialmanager.com/pcompbiol/ and select the 'Submissions Needing Revision' folder to locate your manuscript file.

Please include the following items when submitting your revised manuscript:

* A letter that responds to each point raised by the editor and reviewer(s). You should upload this letter as a separate file labeled 'Response to Reviewers'. This file does not need to include responses to formatting updates and technical items listed in the 'Journal Requirements' section below.

* A marked-up copy of your manuscript that highlights changes made to the original version. You should upload this as a separate file labeled 'Revised Manuscript with Track Changes'.

* An unmarked version of your revised paper without tracked changes. You should upload this as a separate file labeled 'Manuscript'.

If you would like to make changes to your financial disclosure, competing interests statement, or data availability statement, please make these updates within the submission form at the time of resubmission. Guidelines for resubmitting your figure files are available below the reviewer comments at the end of this letter.

As the corresponding author, your ORCID iD is verified in the submission system and will appear in the published article. PLOS supports the use of ORCID, and we encourage all coauthors to register for an ORCID iD and use it as well. Please encourage your coauthors to verify their ORCID iD within the submission system before final acceptance, as unverified ORCID iDs will not appear in the published article. Only the individual author can complete the verification step; PLOS staff cannot verify ORCID iDs on behalf of authors.

We look forward to receiving your revised manuscript.

Kind regards,

Thomas Zdyrski

Guest Editor

PLOS Computational Biology

Feng Fu

Section Editor

PLOS Computational Biology

**Additional Editor Comments (if provided):**

Thank you for implementing the changes recommended by the reviewers. I have a few, minor recommendations:

1. Given the matching color scheme between supplemental figures 4 and 5, I assume figure 5's subfigure columns correspond to cutoff frequency, minimum valid peak height, and maximum valid peak height, respectively. If that is correct, would you please add that information to either the figure or caption of supplemental figure 5? If not, please clarify what the three columns represent.

2. On line 106, there is an upside-down exclamation mark, which should presumably be a less than or less-than-or-equals sign

3. In figure 5's caption, I believe the phrase "(B) the average run length" should be "(B) the average bout length" to match the figure's axis label

4. Please ensure that the supplemental file is submitted without highlighted changes, as it will not be copy-edited

Thank you

**Journal Requirements:**

1) We have noticed that you have a list of Supporting Information legends [S1-S11 Videos] in your manuscript. However, there are no corresponding files uploaded to the submission. Please upload them as separate files with the item type 'Supporting Information'.

2) Please amend your detailed Financial Disclosure statement. This is published with the article. It must therefore be completed in full sentences and contain the exact wording you wish to be published.

1) State what role the funders took in the study. If the funders had no role in your study, please state: "The funders had no role in study design, data collection and analysis, decision to publish, or preparation of the manuscript."

2) If any authors received a salary from any of your funders, please state which authors and which funders..

**Reviewers' comments:**

[NOTE: If reviewer comments were submitted as an attachment file, they will be attached to this email and accessible via the submission site. Please log into your account, locate the manuscript record, and check for the action link "View Attachments". If this link does not appear, there are no attachment files.]

**Figure resubmission:**

-->While revising your submission, we strongly recommend that you use PLOS’s NAAS tool (https://ngplosjournals.pagemajik.ai/artanalysis) to test your figure files. NAAS can convert your figure files to the TIFF file type and meet basic requirements (such as print size, resolution), or provide you with a report on issues that do not meet our requirements and that NAAS cannot fix.

After uploading your figures to PLOS’s NAAS tool - https://ngplosjournals.pagemajik.ai/artanalysis, NAAS will process the files provided and display the results in the "Uploaded Files" section of the page as the processing is complete. If the uploaded figures meet our requirements (or NAAS is able to fix the files to meet our requirements), the figure will be marked as "fixed" above. If NAAS is unable to fix the files, a red "failed" label will appear above. When NAAS has confirmed that the figure files meet our requirements, please download the file via the download option, and include these NAAS processed figure files when submitting your revised manuscript.

**Reproducibility:**

To enhance the reproducibility of your results, we recommend that authors of applicable studies deposit laboratory protocols in protocols.io, where a protocol can be assigned its own identifier (DOI) such that it can be cited independently in the future. Additionally, PLOS ONE offers an option to publish peer-reviewed clinical study protocols. Read more information on sharing protocols at https://plos.org/protocols?utm_medium=editorial-email&utm_source=authorletters&utm_campaign=protocols

# Author comment: Author response to Decision Letter 2

**DOI:** 10.1371/journal.pcbi.1014489.r004

19 Jun 2026

Attachment Submitted filename: PumpKin_ResponseLetter_auresp_2.pdf

# Editor report: Decision Letter 2

**DOI:** 10.1371/journal.pcbi.1014489.r005

**Authors:** Feng Fu, Thomas Zdyrski

22 Jun 2026

Dear Dr. Lu,

We are pleased to inform you that your manuscript 'PumpKin: A machine-learning pipeline for automatically tracking localized kinematics in freely moving C. elegans' has been provisionally accepted for publication in PLOS Computational Biology.

Before your manuscript can be formally accepted you will need to complete some formatting changes, which you will receive in a follow up email. A member of our team will be in touch with a set of requests.

Please note that your manuscript will not be scheduled for publication until you have made the required changes, so a swift response is appreciated.

IMPORTANT: The editorial review process is now complete. PLOS will only permit corrections to spelling, formatting or significant scientific errors from this point onwards. Requests for major changes, or any which affect the scientific understanding of your work, will cause delays to the publication date of your manuscript.

Should you, your institution's press office or the journal office choose to press release your paper, you will automatically be opted out of early publication. We ask that you notify us now if you or your institution is planning to press release the article. All press must be co-ordinated with PLOS.

Thank you again for supporting Open Access publishing; we are looking forward to publishing your work in PLOS Computational Biology.

Best regards,

Thomas Zdyrski

Guest Editor

PLOS Computational Biology

Feng Fu

Section Editor

PLOS Computational Biology

***********************************************************

# Editor report: Acceptance letter

**DOI:** 10.1371/journal.pcbi.1014489.r006

**Authors:** Feng Fu, Thomas Zdyrski

PCOMPBIOL-D-26-00134R2

PumpKin: A machine-learning pipeline for automatically tracking localized kinematics in freely moving C. elegans

Dear Dr Lu,

I am pleased to inform you that your manuscript has been formally accepted for publication in PLOS Computational Biology. Your manuscript is now with our production department and you will be notified of the publication date in due course.

The corresponding author will soon be receiving a typeset proof for review, to ensure errors have not been introduced during production. Please review the PDF proof of your manuscript carefully, as this is the last chance to correct any errors. Please note that major changes, or those which affect the scientific understanding of the work, will likely cause delays to the publication date of your manuscript.

Soon after your final files are uploaded, unless you have opted out, the early version of your manuscript will be published online. The date of the early version will be your article's publication date. The final article will be published to the same URL, and all versions of the paper will be accessible to readers.

For Research, Software, and Methods articles, you will receive an invoice from PLOS for your publication fee after your manuscript has reached the completed accept phase. If you receive an email requesting payment before acceptance or for any other service, this may be a phishing scheme. Learn how to identify phishing emails and protect your accounts at https://explore.plos.org/phishing.

Thank you again for supporting PLOS Computational Biology and open-access publishing. We are looking forward to publishing your work!

With kind regards,

Sharmila Kamatchi

PLOS Computational Biology | Carlyle House, Carlyle Road, Cambridge CB4 3DN | United Kingdom ploscompbiol@plos.org | Phone +44 (0) 1223-442824 | ploscompbiol.org | @PLOSCompBiol
